# DFINE-PFM: a real-time instance segmentation network for potato harvester conveyor belts

**DOI:** 10.3389/fpls.2026.1906894

**Published:** 2026-08-04

**Authors:** Qiyu Zhang, Xinping Mo, Huan Zhang, Wenjing Li, Guangfei Liu, Ranbing Yang, Zhiguo Pan

**Affiliations:** 1 School of Mechanical and Electrical Engineering, Qingdao Agricultural University, Qingdao, China; 2 Department of Information Engineering, Shandong Foreign Trade Vocational College, Qingdao, China; 3 School of Mechanical and Electrical Engineering, Hainan University, Haikou, China

**Keywords:** conveyor belt quality control, image-based yield estimation, mechanized potato harvesting, real-time instance segmentation, soil clod discrimination

## Abstract

Potato is an important staple crop worldwide, and accurate tuber detection is essential for intelligent harvesting, yield estimation, and quality management. During mechanized harvesting, potatoes are conveyed with large amounts of soil clods on conveyor belts. The similar appearance between soil-covered tubers and clods makes accurate identification and separation difficult under practical operating conditions. Existing methods are often limited by scale variation, object occlusion, irregular boundaries, and complex backgrounds, restricting their application in real-time harvesting systems.This study proposes DFINE-PFM, an end-to-end instance segmentation framework for automatic potato–clod discrimination on harvesting conveyor belts. The framework incorporates four targeted improvements: a multi-scale feature diffusion module to enhance feature representation, a parallel large-kernel decomposition module to improve boundary extraction, a heterogeneous multi-branch convolution module to capture multi-granularity features with reduced redundancy, and an asymmetric receptive field convolution module to improve localization of small and irregular objects. A self-collected conveyor belt dataset under practical harvesting conditions was used for evaluation. DFINE-PFM achieved Precision, Recall, F1-score, and mAP values of 98.80%, 98.70%, 98.75%, and 98.03%, respectively, with an inference time of 43.41 ms. Compared with the baseline model, it improved Precision and mAP by 6.20 and 5.60 percentage points, respectively. Deployment experiments demonstrated stable real-time performance at 20 FPS under conveyor speeds of 0.1–0.6 m/s. The proposed method provides an effective solution for automated potato–clod separation and intelligent harvesting quality monitoring.

## 1 Introduction

Potatoes are the world’s fourth-largest crop, with an annual production exceeding 374 million tons, and as such, they play a significant role in food security, poverty alleviation, and wealth creation. With the shrinking labor force and rising labor costs, automation in the potato industry has become an inevitable trend. Machine vision enhances machines’ environmental perception capabilities (Xu and Lu, 2024). Renowned for its efficiency, speed, and non-contact nature, this technology has been widely adopted for agricultural product quality assessment (Bi et al., 2019; Xie et al., 2020; Mao et al., 2024; Zhang et al., 2025; Zhang et al., 2026).

Accurately identifying and segmenting potatoes in complex environments is a primary task and a critical step in the automation of the potato industry through image processing. Researchers have implemented machine vision systems in various ways to automate potato recognition across different applications. Verk et al. (2025) addressed the need for efficient segmentation of multiple potato tubers simultaneously in potato sorting systems by adopting the Mask R-CNN architecture to achieve precise instance segmentation through the generation of pixel-level segmentation masks. They systematically evaluated the model’s performance under different parameter settings, achieving a segmentation accuracy (mAP) of 87.8%, demonstrating the effectiveness and feasibility of Mask R-CNN in industrial potato sorting production processes. However, Mask R-CNN is a two-stage framework where feature extraction and mask prediction are executed sequentially, resulting in slow inference speeds that struggle to meet real-time segmentation requirements. Its RoIAlign operation has limited ability to detect the boundaries of small targets and irregularly shaped tubers, leading to a significant drop in segmentation mask accuracy in scenarios where potatoes occlude or stack on top of one another. Furthermore, the model’s large number of parameters makes it prone to overfitting on small-scale agricultural datasets. Zhao et al. (Li et al., 2025) addressed the issues of low efficiency and high cost in manual potato quality inspection, as well as the inability of single-task deep learning methods to achieve multidimensional quality assessment. They proposed the multi-task learning network YOLO-MTP, which simultaneously introduces an edibility detection branch and a surface defect semantic segmentation branch based on the YOLOv8s framework. By enhancing feature extraction capabilities through dynamic convolutions and an efficient multi-scale attention mechanism, the method achieves an object detection accuracy of 96.7%, and a mean Intersection over Union (mIoU) of 80.03% across six defect segmentation tasks. Although this method introduces a semantic segmentation branch based on YOLOv8s, semantic segmentation cannot distinguish between different instances within the same category, making it impossible to achieve instance-level precise segmentation in scenes with dense clusters of potato tubers; in multi-task learning, there is a gradient conflict between detection and segmentation objectives, which limits segmentation accuracy; simultaneously, as this method is designed for defect detection, it pays insufficient attention to the fine segmentation of the overall potato contour. Li et al. (2022) constructed a three-stage ensemble framework integrating instance segmentation, classification, and semantic segmentation for detecting potato leaf diseases in complex backgrounds. In the first stage, Mask R-CNN was used to segment leaves from complex backgrounds (AP reached 81.87%); in the third stage, three semantic segmentation models—UNet, PSPNet, and DeepLabV3+ were compared (the second stage performed classification). Among them, UNet achieved an mIoU of 89.91% and an MPA of 94.24%, validating the effectiveness of the multi-stage segmentation framework in the accurate identification of potato leaf diseases. However, the three-stage serial framework is complex, and errors accumulate and propagate through each stage. The overall accuracy is constrained by the least accurate stage. Since the three models are trained independently, end-to-end optimization is not feasible, rendering the training process cumbersome.

The Match-Aware Loss (MAL) proposed by DFINE is specifically optimized to address low-quality matches (Huang et al., 2025), which is particularly important for potato segmentation tasks. Potato images often contain occlusions, irregular shapes, and overlapping objects, which generate a large number of low-quality match samples. By directly incorporating the IoU between the matched query and the ground truth into the loss function, MAL imposes a heavier penalty on low-quality matches. Compared to traditional VFL loss, this approach more effectively utilizes these challenging samples, thereby improving the detection accuracy of irregular potato tuber boundaries. Furthermore, DFINE offers a significant advantage in the trade-off between accuracy and speed, making it suitable for real-time applications. Therefore, to address the aforementioned issues, this paper proposes an innovative and efficient detection framework, DFINE-PFM, based on DFINE. The novelty of DFINE-PFM lies in the task-driven co-design and integration of its components for real-time tuber–clod instance segmentation, rather than in proposing new low-level operators in isolation. This framework is specifically tailored for the real-time detection and counting of potatoes on the conveyor belt of a potato harvester. Real-time detection and counting of potatoes on the conveyor belt facilitates real-time yield estimation and the automated removal of debris such as soil clods, which holds significant practical value. The framework introduces three key algorithmic enhancements: (1) Partial Convolution (PConv), which reduces redundant computations and memory access to extract spatial features more efficiently, thereby lowering the computational load of the model and enabling more efficient deployment on edge devices such as the harvester. (2) Feature Focused Diffusion Pyramid Network (FDPN), a novel feature processing framework that significantly enhances the contextual information representation of feature maps through a custom feature focusing module (FocusFeature) and feature diffusion. Through deep integration of cross-scale features, this framework ensures that feature maps at every scale not only retain local spatial details but also incorporate global semantic information, providing more discriminative and robust feature representations for subsequent object detection and classification tasks. Furthermore, within the feature focusing module of FDPN, we replace the conventional multi-scale feature extraction method with the IDWB feature extraction approach, which introduces dynamic weight allocation to strengthen the emphasis on salient features. (3) MS-Block enhances the real-time object detector’s ability to extract multi-scale features while ensuring fast inference performance. To achieve precise segmentation of potato contours rather than mere potato detection, we replace the object detection decoder (DFINETransformer) with an instance segmentation decoder (DFINESegTransformer). Building upon DFINE’s fine-grained distribution optimization mechanism, DFINESegTransformer introduces a pixel-level segmentation head that outputs high-resolution instance segmentation masks at a segmentation stride of 4. This enables a task upgrade from object detection to instance segmentation, supporting precise, independent contour segmentation for each individual potato.

## 2 Experimental preparation

### 2.1 Data collection and preprocessing

**Figure 1:**
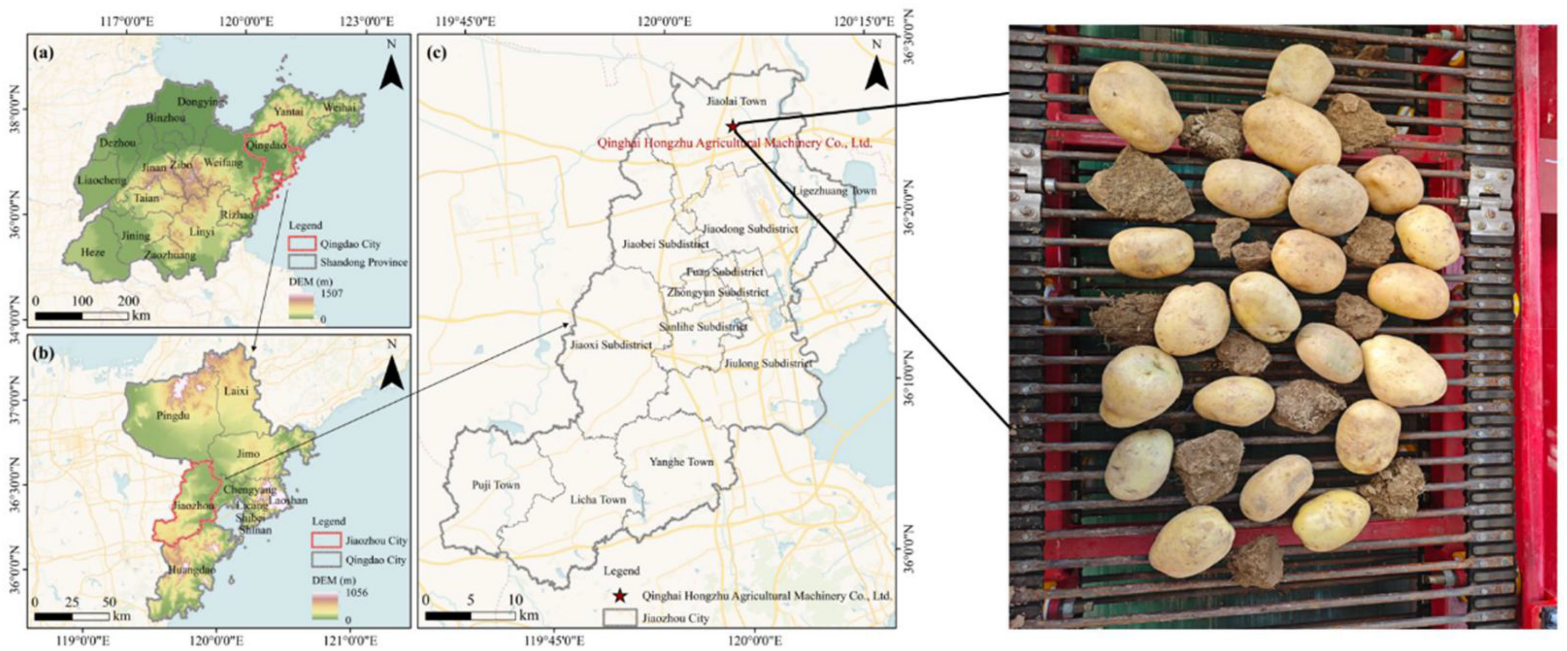
Experimental site and image acquisition during mechanized potato harvesting. **(a)** Location of Shandong Province; **(b)** experimental area in Qingdao City; and **(c)** harvesting site and conveyor belt images of potatoes and soil clods.

The images used in this study were sourced from a self-compiled potato dataset. The captured images consist of potatoes and soil clods, with a resolution of 4096×3072 pixels. The images were collected in September 2025 using the Hongzhu 4U-170 large-scale potato harvester and the Yatai Machinery 4UQL-160 potato combine harvester, respectively. The collection locations are shown in the **Figure 1**. To account for varying detection heights, the experimenters used a height-adjustable tripod to position a Redmi K70 smartphone directly above the conveyor belt at a distance of 40–50 cm, capturing images of potatoes and soil clods at different heights. To simulate real-world conditions during potato harvester operation, a small number of potatoes were deliberately arranged in partially overlapping configurations to simulate occlusion, and a small amount of soil was applied to the potato surfaces. The layout was randomly reconfigured after every five sets of images were captured. Using the aforementioned imaging setup, a total of 835 images of potatoes and soil clods were collected and subsequently annotated for training and evaluation.

To ensure consistency in the input data and avoid errors caused by varying image sizes, all images were uniformly cropped. Additionally, to simulate the need for shots taken from different angles, the cropped images were randomly rotated. After completing image preprocessing, the Labelme tool was used to annotate the potatoes and soil clods, locating and marking their positions and contours.

**Figure 2:**
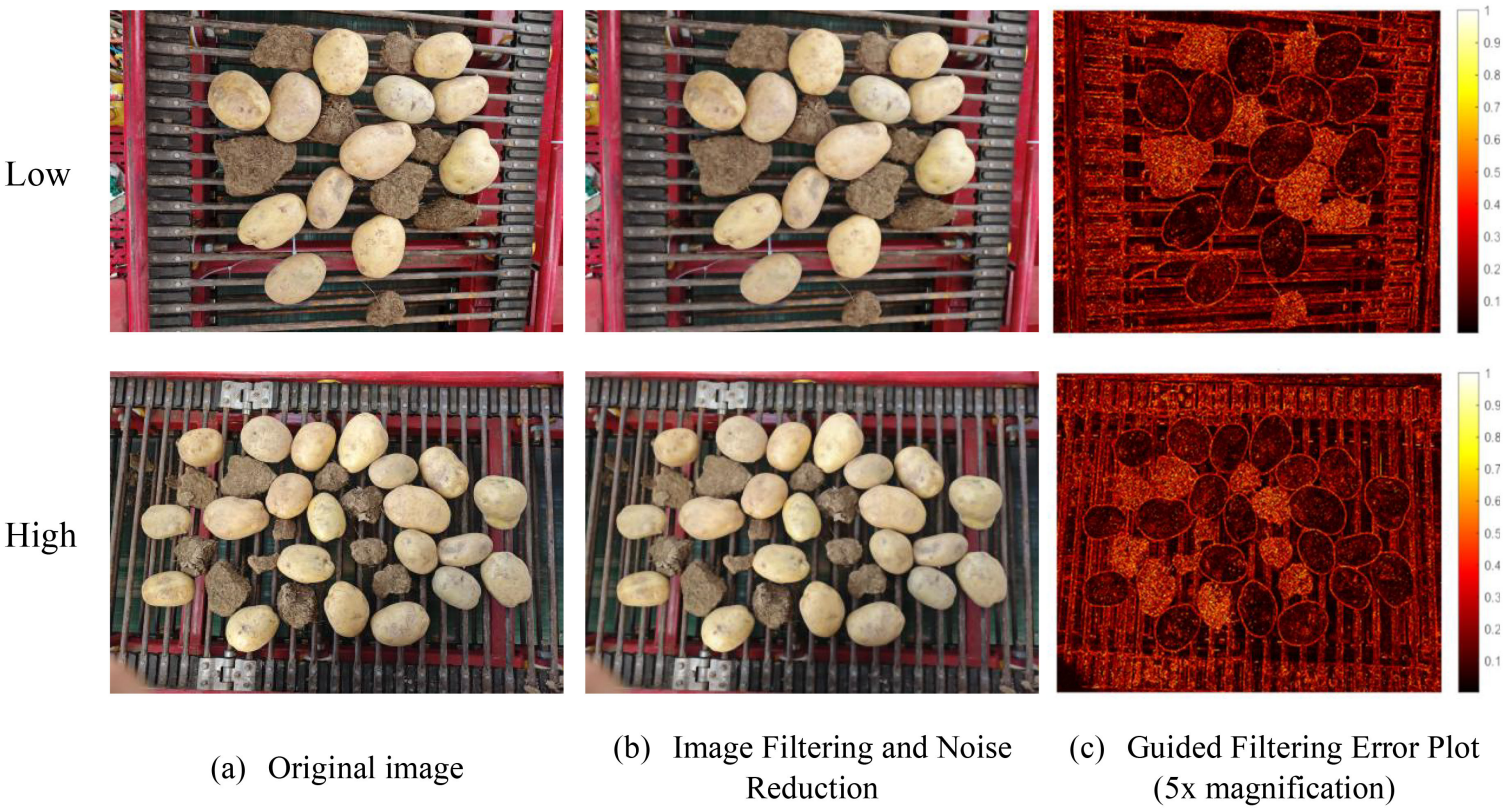
Illustration of the noise reduction effect achieved through directional filtering. **(a)** Original image. **(b)** Image filtering and noise reduction. **(c)** Guided filtering error plot (5x magnification).

After data preprocessing, the dataset was split. The dataset for this study consists of 835 images, divided into a training set (585 images, 70%), a validation set (166 images, 20%), and a test set (84 images, 10%) following a 7:2:1 ratio. During the first 50% of training iterations, the model was trained with Mosaic augmentation and MixUp augmentation. Mosaic augmentation involves randomly selecting four potato images during model training, then randomly cropping, flipping, rotating, color jittering, and scaling them to stitch them into a new image. MixUp augmentation involves linearly interpolating two images and their corresponding labels at a randomly sampled ratio, which effectively increases the number of targets in each image. In addition, we applied directional filtering-based denoising to all collected images. The denoised images were incorporated into the original dataset to construct the final experimental dataset, thereby increasing the diversity of the training data. The training set now consists of 2,170 images, while the test set and validation set remain unchanged. The denoising results are shown in **Figure 2**.

To characterize the diversity of the dataset, images were acquired with two different harvesters (the Hongzhu 4U-170 and the Yatai Machinery 4UQL-160) at multiple field sites, and captured with the camera positioned 40–50 cm above the belt at varying heights and angles. The samples span different degrees of soil cover on the tuber surface, different tuber–clod layouts including deliberately arranged partial occlusions, and a range of soil-clod sizes and morphologies. Collecting larger, multi-seasonal, multi-regional datasets remains part of our future work.

To preclude data leakage, the split into training, validation, and test subsets was performed at the level of the 835 original images before any augmentation was applied. Mosaic augmentation, MixUp augmentation, and directional-filter denoising were applied only to the training subset; no augmented, denoised, or otherwise source-derived version of any validation or test image appears in the training set. The test set therefore contains only original, unaugmented, previously unseen images, and the reported test metrics reflect genuine generalization rather than memorization.

### 2.2 Preparation of experimental equipment

The development platform used in this study is configured as follows: an NVIDIA GeForce RTX 4090 Ti graphics processing unit (GPU) with 24 GB of memory, PyCharm as the integrated development environment (IDE), Python as the programming language, and Linux as the operating system. The deep learning models were built using the PyTorch framework with CUDA 12.9 for GPU acceleration. **Table 1** provides a summary of all details related to the experimental setup. Configuration as shown in the **Table 1**.

**Table 1:** Experimental environment configuration.

Category	Configuration
CPU	AMD EPYC 7K62 48-Core Processor
GPU	Nvidia 4090-24G
System environment	Linux 6.1.0-33-amd64
Framework	Pytorch2.1.0
Programming	Python3.10.14

**Figure 3:**
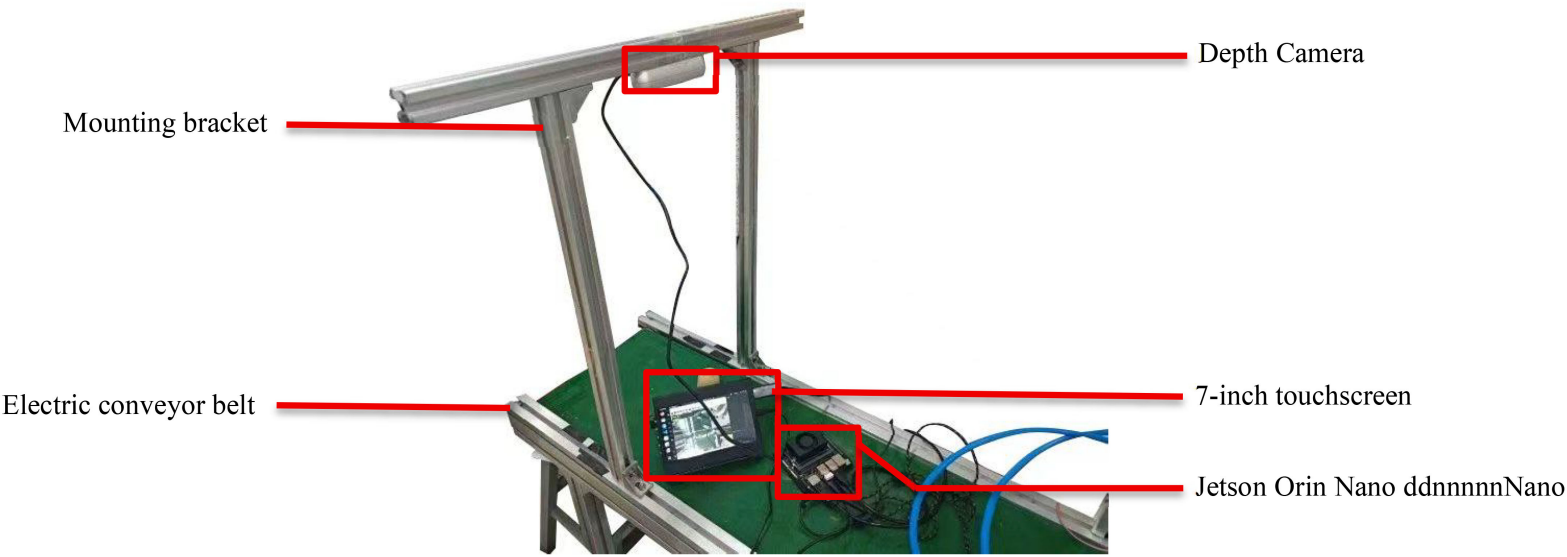
Experimental platform.

To test the model in a real-world deployment scenario, we built an experimental platform consisting of an electric conveyor belt, a height-adjustable mounting bracket, an Intel RealSense D456 depth camera, an NVIDIA Jetson Orin Nano Super 8GB, and a 7-inch touchscreen. The depth camera is mounted on the height-adjustable bracket positioned above the conveyor belt, capturing real-time images of potatoes and soil clods on the motorized conveyor belt. The Jetson Orin Nano is used for model deployment, real-time inference, and control of the depth camera, and the final segmentation results are displayed on the touchscreen. The experimental setup is shown in **Figure 3**.

## 3 The DFINE-PFM segmentation model

Convolution layers form the fundamental computational units of CNNs, where kernel size and quantity determine the effective receptive field governing local information aggregation. Given severe occlusion between potato instances and between potatoes and soil clods, the segmentation model requires strong multi-scale feature extraction capabilities and a large receptive field to capture boundary features and aggregate global contextual information.

**Figure 4:**
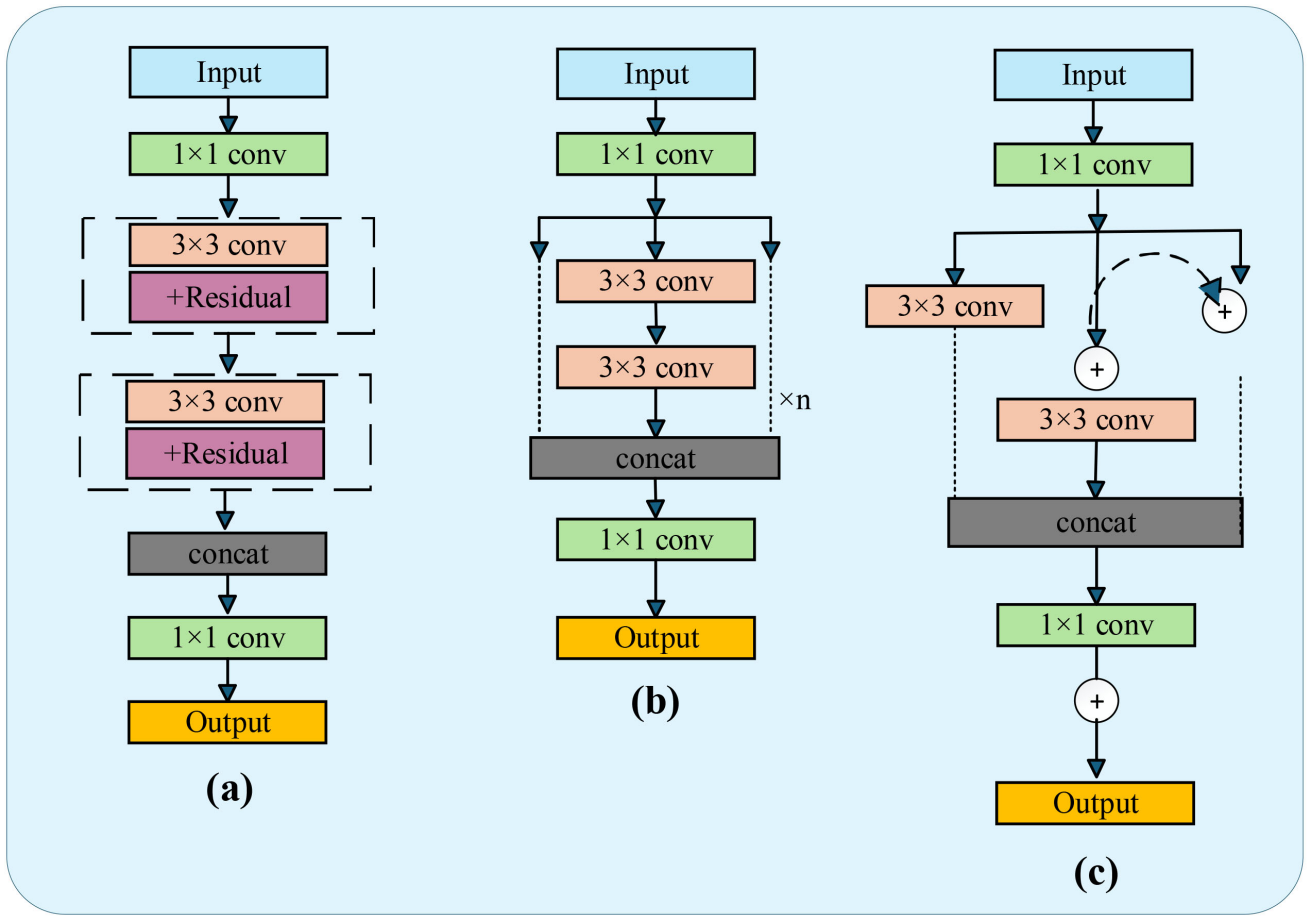
Common convolutional block architectures **(a)** CSP block **(b)** ELAN block **(c)** Res2Net block.

As shown in **Figure 4**, mainstream modules including CSP and ELAN suffer from feature redundancy and structural homogeneity, limiting multi-scale detection performance. Res2Net enhances feature diversity through hierarchical multi-branch structures but introduces “contaminant spatial information” that degrades localization accuracy. The original MSBlock addresses these issues through an inverted bottleneck structure and grouped accumulation mechanism, yet still exhibits three limitations in the conveyor belt scenario: (1) homogeneous kernel configuration [1, 3, 3] causes inter-branch feature redundancy, failing to simultaneously capture fine-grained textures and coarse-grained contours; (2) unidirectional feature propagation restricts information exchange between shallow and deep branches; (3) absence of a module-level residual connection causes loss of non-directional semantic information during channel compression. Therefore, this study proposes MSBlock_Plus to address these limitations while preserving the core MSBlock design philosophy.

### 3.1 Standard DFINE model

In this study, the lightweight model DFINE-n was selected for deployment on the Jetson Orin Nano. The system consists of three core components: a backbone network, an encoder network, and a Transformer-based decoder. The backbone network extracts multi-scale feature representations from RGB images using a hierarchical convolutional approach. The encoder network fuses and enhances the multi-scale features extracted by the backbone. This method employs a hybrid encoder architecture that combines the multi-head self-attention mechanism of the Transformer with a convolutional feature pyramid network, effectively integrating low-level local spatial details with high-level global semantic information. The Transformer-based decoder classifies and localizes objects in an end-to-end manner. During training, it employs the Hungarian algorithm to establish a one-to-one assignment between predicted and ground-truth bounding boxes, eliminating the need for non-maximum suppression post-processing, thereby reducing inference latency and enhancing detection stability (Carion et al., 2020).

The architecture of the DFINE backbone network consists of a StemBlock initial feature extraction module and four HG_Stage hierarchical feature extraction modules. The StemBlock performs initial convolution operations and strided downsampling on the input images, while the HG_Stage modules are composed of multiple stacked lightweight convolutional layers and incorporate a Squeeze-and-Excitation channel attention mechanism to adaptively enhance important channel features and suppress redundant information (Hu et al., 2018).

### 3.2 Enhanced DFINE architecture

**Figure 5:**
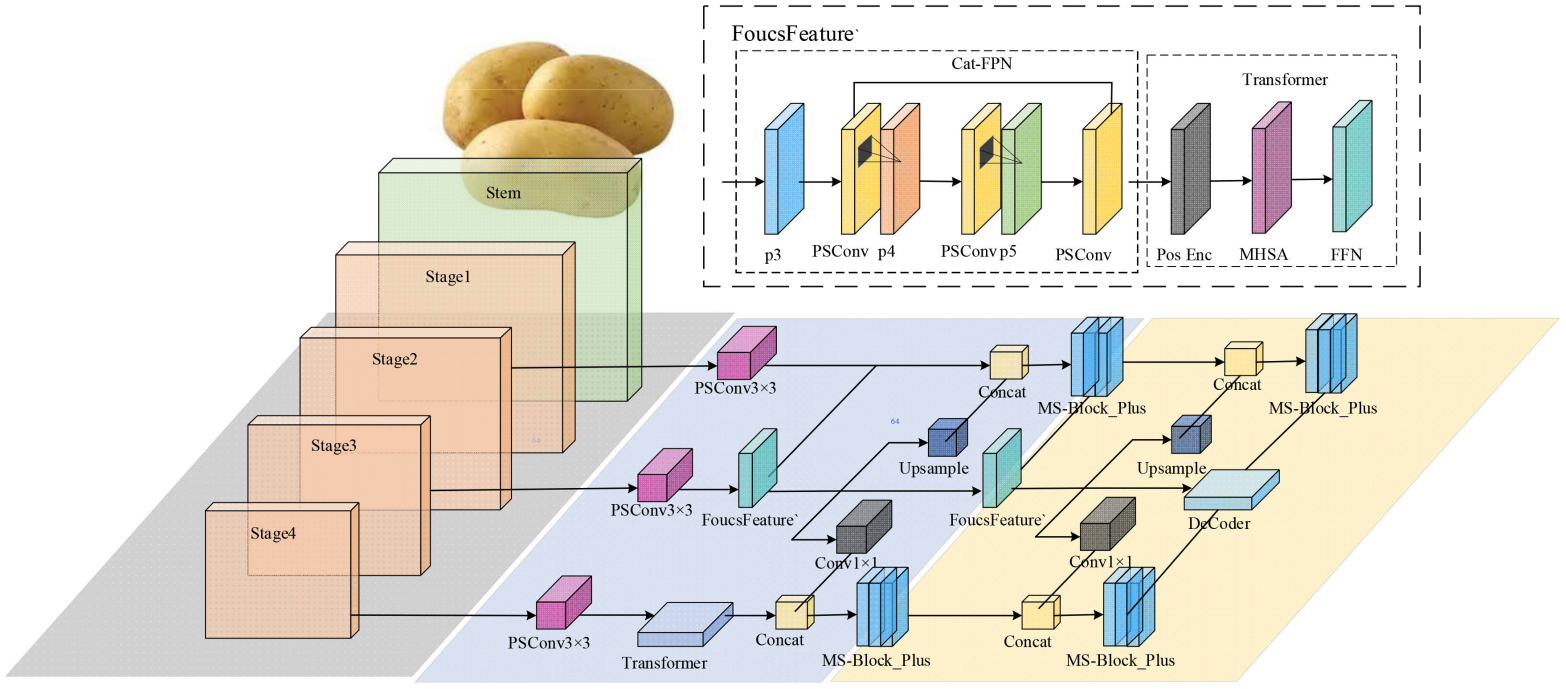
DFINE-PFM network architecture.

Based on the Transformer detection framework, this study addresses the inherent limitations of its encoder during the multi-scale feature fusion stage by proposing the Feature Focused Diffusion Pyramid Network (FDPN), which systematically restructures the encoder architecture: First, building upon the original dual-scale P4 and P5 layers, we introduce an additional P3 layer for high-resolution features, establishing a three-scale parallel input structure (Lin et al., 2017a). This enables the Transformer encoder to capture global semantic information while preserving fine-grained spatial details at the object boundaries. Second, to address the semantic inconsistency between the Transformer encoder’s output features and the multi-scale convolutional features from the backbone, we design the FocusFeature module to replace the simple concatenation operation. This module adopts an Inception-inspired multi-branch parallel depthwise convolutional architecture. Through multi-scale receptive field kernels, it adaptively extracts cross-scale spatial patterns and semantic features (Szegedy et al., 2015). By combining depthwise separable convolutions with 1×1 dimension-reduction convolutions, it enhances feature expressiveness while effectively controlling computational overhead. Furthermore, to improve the core feature enhancement structure of the FocusFeature module, we adopt the InceptionDWBlock—a channel-split multi-branch depthwise decomposition following the Inception design family—as the feature-enhancement unit, replacing the original combination of independent depthwise convolutions. While the block itself derives from prior work, its role here, serving as the intra-FocusFeature enhancement unit that enables cross-branch information exchange across receptive fields, is what benefits the soil-covered boundary segmentation task (Howard et al., 2017). Through its multi-branch parallel convolutional architecture, the InceptionDWBlock achieves collaborative feature extraction across multiple receptive fields within a single module. This effectively overcomes the limitations of the original approach, where convolutional branches operated independently and lacked information exchange between branches. Without altering the overall network topology, it significantly enhances the expressive power and computational efficiency of three-scale fused features, and is named FocusFeature_IDWB. Third, the 1×1 ConvNormLayer_fuse in the backbone feature compression stage is replaced with a 3×3 PSConv. This introduces richer local spatial context modeling while maintaining channel dimensionality alignment, providing richer feature inputs for subsequent global modeling by the Transformer. Finally, the three RepNCSPELAN4 feature enhancement modules in the encoder are uniformly replaced with MSBlock. The latter adopts a multi-branch parallel convolutional architecture to achieve adaptive enhancement of cross-scale features with a lighter parameter size, effectively alleviating the limitations of fixed receptive field structures when processing potato tubers of different sizes. The improved model structure is shown in **Figure 5**.

### 3.3 Structure of MSBlock_Plus

Convolution layers form the fundamental computational units of CNNs, where kernel size and quantity determine the effective receptive field governing local information aggregation. Given severe occlusion between potato instances and between potatoes and soil clods, the segmentation model requires strong multi-scale feature extraction capabilities and a large receptive field to capture boundary features and aggregate global contextual information.

As shown in **Figure 4**, mainstream modules including CSP (Wang et al., 2020) and ELAN (Wang et al., 2023) suffer from feature redundancy and structural homogeneity, limiting multi-scale detection performance. Res2Net enhances feature diversity through hierarchical multi-branch structures but introduces “contaminant spatial information” that degrades localization accuracy (Gao et al., 2021). The original MSBlock addresses these issues through an inverted bottleneck structure and grouped accumulation mechanism, yet still exhibits three limitations in the conveyor belt scenario: (1) homogeneous kernel configuration [1, 3, 3] causes inter-branch feature redundancy, failing to simultaneously capture fine-grained textures and coarse-grained contours; (2) unidirectional feature propagation restricts information exchange between shallow and deep branches; (3) absence of a module-level residual connection causes loss of non-directional semantic information during channel compression. Therefore, this study proposes MSBlock_Plus to address these limitations while preserving the core MSBlock design philosophy.

**Figure 6:**
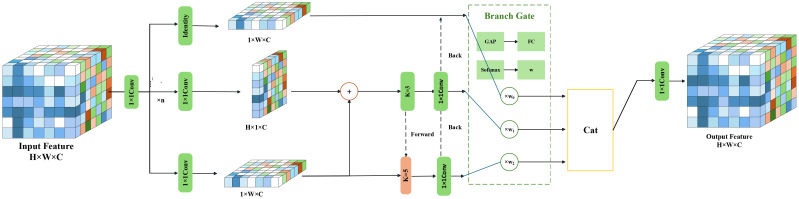
MSBlock_Plus module structure.

The module structure of MSBlock_Plus is shown in **Figure 6**. Similar to the original MSBlock, input features are first expanded to n×c channels via a 1×1 convolution, then divided into n groups for multi-branch convolution at different scales. Four targeted improvements are incorporated.

First, heterogeneous kernel configuration [1, 3, 5]. The original homogeneous configuration [1, 3, 3] causes inter-branch feature redundancy. MSBlock_Plus assigns distinct kernel sizes to each branch: Identity mapping for feature preservation, 3×3 kernels for fine-grained local features such as potato surface textures and clod micro-crack edges, and 5×5 kernels for coarse-grained features such as tuber contour curvature and clod boundary morphology. The two convolutional branches are thus complementary, enabling multi-granularity perception with negligible additional overhead via depthwise convolution (Tan and Le, 2019).

Second, bidirectional progressive aggregation. The original unidirectional flow (0→1→2) prevents shallow branches from accessing deep semantic representations. MSBlock_Plus adds a reverse pass (2→1→0) following the forward pass, enabling each branch to integrate contextual information from all other branches and ensuring full multi-scale information sharing across the network (Lin et al., 2017b). The principle is as shown in Equations (1, 2).

(1) $$f_{i}^{fwd}=\{\begin{array}{ll} s_{0} & i=0\\ B_{k_{i}}(s_{i}+f_{i-1}^{fwd}) & i\geq 1 \end{array}$$

(2) $$f_{i}^{bwd}=\{\begin{array}{ll} f_{2}^{fwd} & i=2\\ f_{i}^{fwd}+f_{i+1}^{bwd} & i<2 \end{array}$$

Third, adaptive branch importance gating. Rather than fixed equal-weight concatenation, a lightweight gating mechanism computes per-branch scalar weights via global average pooling, a two-layer MLP (compression ratio 8), and Softmax normalization. This allows dynamic adjustment of branch contributions based on input content, with total parameter overhead of only n×c/r parameters (Hu et al., 2018). The principle is as shown in Equation (3).

(3) $${\hat{f}}_{i}=f_{i}^{bwd}\cdot w_{i},\;w_{i}=\text{Softmax}{\left(W_{2}\cdot \text{ReLU}(W_{1}\cdot \text{GAP}(g))\right)}_{i}$$

Fourth, module-level residual connection. The absence of residual connections in the original MSBlock risks losing non-directional semantic information during the 2:1 channel compression at encoder fusion nodes. A 1×1 projected residual pathway is introduced, bypassing the multi-branch convolution to preserve global contextual features and improve gradient flow during training. The principle is as shown in Equation (4).

(4) $$Y={\text{Conv}}_{1\times 1}(\text{Concat}({\hat{f}}_{0},{\hat{f}}_{1},{\hat{f}}_{2}))+{\text{Conv}}_{1\times 1}(X)$$

In summary, MSBlock_Plus achieves comprehensive multi-scale feature capture through these four synergistic improvements, simultaneously enabling accurate localization of coarse-grained potato contours and precise segmentation of fine-grained surface details in complex conveyor belt environments, while maintaining computational efficiency.

### 3.4 Structure of PSConv

Due to the high computational complexity of the DFINE architecture, fine-grained spatial features of small objects are progressively lost after multi-layer convolution operations, primarily due to progressive resolution reduction and the attenuation of fine-grained spatial details. Consequently, when using traditional convolutions to segment the irregular contours of potatoes, some of the fine-grained boundary features are inevitably lost. To address this issue, this study adopts the Windmill-shaped Convolution (PSConv) as a replacement for the standard convolutions in the lower layers of the backbone network. PSConv better aligns with the Gaussian spatial distribution of pixels in small and low-contrast targets, thereby enhancing local feature extraction capability and significantly enlarging the effective receptive field, while introducing only minimal additional parameters. Through its windmill-shaped receptive field structure, PSConv strengthens the model’s sensitivity to fine-grained local features of small objects while suppressing the interference of background clutter and irrelevant contextual information (Yang et al., 2025).

**Figure 7:**
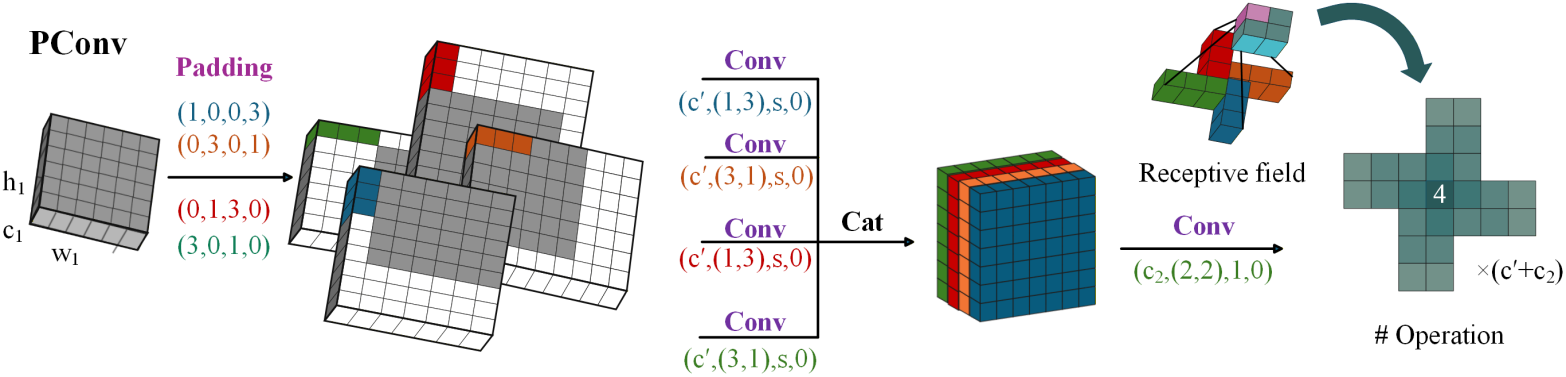
PSConv module structure.

**Figure 7** shows the architecture of PSConv. As shown in the figure, the PSConv module first performs four-branch parallel convolution operations on the input feature map X of size (H_1_ × W_1_ × C_1_), where H_1_, W_1_, and C_1_ denote the height, width, and number of channels, respectively. It generates directional convolution kernels in four asymmetric directions through asymmetric padding to process different spatial regions of the feature map, with each convolution branch configured with direction-specific asymmetric padding parameters. The output feature maps from all four branches are then concatenated along the channel dimension. Finally, the concatenated feature maps are normalized, and a 1×1 pointwise convolution is applied to adjust the output channel dimension. The principle is as shown in Equation (5).

(5) $$X^{\left(h^{\prime },w^{\prime },c^{\prime }\right)}=S\text{iLU}(BN(C\text{onv}2\text{d}({\text{C}}^{\text{out}}\text{k},\text{s},\text{p},)))$$

Batch Normalization (BN) and the Sigmoid Linear Unit (SiLU) activation function are applied after each convolution. In the equation, Conv2d represents two-dimensional convolution, meaning that the convolution kernel slides across a two-dimensional feature map, performing element-wise multiply-accumulate operations between the kernel weights and the corresponding input values at each sliding position. C_out represents the number of output channels; k represents the convolution kernel size; s represents the stride; p represents the padding configuration, where Padding(left, right, top, bottom) specifies the number of pixels padded along the left, right, top, and bottom borders, respectively. The output features from the four-branch directional convolutions are concatenated along the channel dimension to obtain a feature map with a channel dimension of 4c′. The principle is as shown in Equations (6, 7).

(6) $$h^{\prime }=\frac{h_{1}}{s}+1,w^{\prime }=\frac{w_{1}}{s}+1,c^{\prime }=\frac{c2}{4}$$

(7) $${\mathrm{X'}}^{\left(h^{\prime },w^{\prime },4c^{\prime }\right)}=Cat(X_{1},X_{2},X_{3},X_{4})$$

Here, h′, w′, and c′ denote the height, width, and number of channels of the intermediate feature maps, respectively; c_2_ denotes the number of output channels of the PSConv module; and X_1_–X_4_ denote the output feature maps of the four directional convolution branches, respectively.

### 3.5 Structure of FDPN

**Figure 8:**
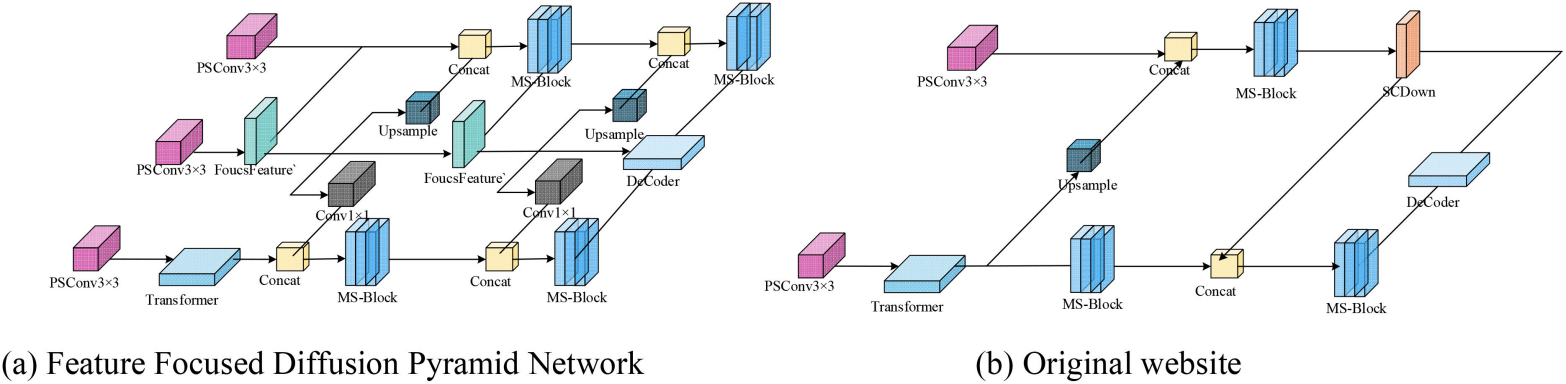
Comparison of network structures. **(a)** Feature focused diffusion pyramid network. **(b)** Original website.

Traditional FPNs suffer from information loss during cross-scale feature propagation, leading to a high miss rate in the detection of small potato instances (Lin et al., 2017a). Additionally, potatoes often overlap or partially occlude one another, imposing stringent requirements on inter-instance discriminability in instance segmentation. To address these issues, we introduce the Feature Focused Diffusion Pyramid Network (FDPN), which introduces a systematic multi-scale feature processing strategy. Through a custom feature focusing module (FocusFeature) and a feature diffusion mechanism, this network significantly enhances the contextual representational capacity of multi-scale feature maps. Through the deep integration of cross-scale features, this framework ensures that feature maps at every scale not only retain local spatial details but also incorporate global semantic information, thereby providing richer multi-scale representations compared to traditional network architectures, as illustrated in **Figure 8**. Compared with representative feature-fusion structures, FDPN differs in the following ways. FPN propagates semantics through a single top-down additive path, which loses fine detail for small tubers. PANet adds a complementary bottom-up path to shorten the information route, but still relies on element-wise addition and a fixed fusion order. BiFPN introduces learnable weights and repeated bidirectional blocks, yet fuses features of adjacent scales in a pairwise manner. In contrast, FDPN performs a focus-then-diffuse operation: the FocusFeature module first aggregates all three scales jointly through parallel multi-receptive-field depthwise branches rather than pairwise addition, and the two-stage diffusion mechanism then redistributes this jointly focused representation across scales. This joint-focus design is better suited to the strong scale variation and boundary ambiguity between soil-covered tubers and adhering clods. This provides highly discriminative and semantically enriched feature representations for subsequent instance segmentation tasks.

The Feature Focusing Module is the core innovative unit of FDPN, specifically designed to process feature inputs at three different scales (Szegedy et al., 2015). Internally, this module employs an Inception-inspired multi-branch architecture that extracts cross-scale spatial patterns and semantic features across various receptive fields through a set of parallel depthwise convolutional kernels with kernel sizes of {5, 7, 9, 11}. Furthermore, the module combines depthwise separable convolutions (Howard et al., 2017) and pointwise convolutions (1×1 convolutions) to enhance feature expressiveness while effectively controlling computational overhead. Finally, 1×1 convolutions are applied to compress the channel dimension and integrate the features, ensuring that the output feature maps possess rich contextual information while maintaining dimensional consistency.

FDPN’s feature diffusion mechanism efficiently propagates semantically enriched multi-scale features across detection scales through hierarchical feature propagation pathways that integrate both top-down and bottom-up information flows (Liu et al., 2018). In the first stage of the network, the output of the feature focusing module is fused with high-resolution and low-resolution feature maps via downsampling and upsampling operations, forming an initial multi-scale feature representation. In the second stage, the network further employs a secondary feature focusing and deep feature enhancement module, specifically the Cross-Stage Partial with Two convolutions (C2f) module (Jocher et al., 2023), to achieve deep feature integration and comprehensive diffusion of contextual information. This two-stage feature diffusion strategy effectively mitigates the information loss caused by scale differences in traditional feature pyramid networks, significantly enhancing the semantic consistency and representational capacity of multi-scale features.

**Figure 9:**
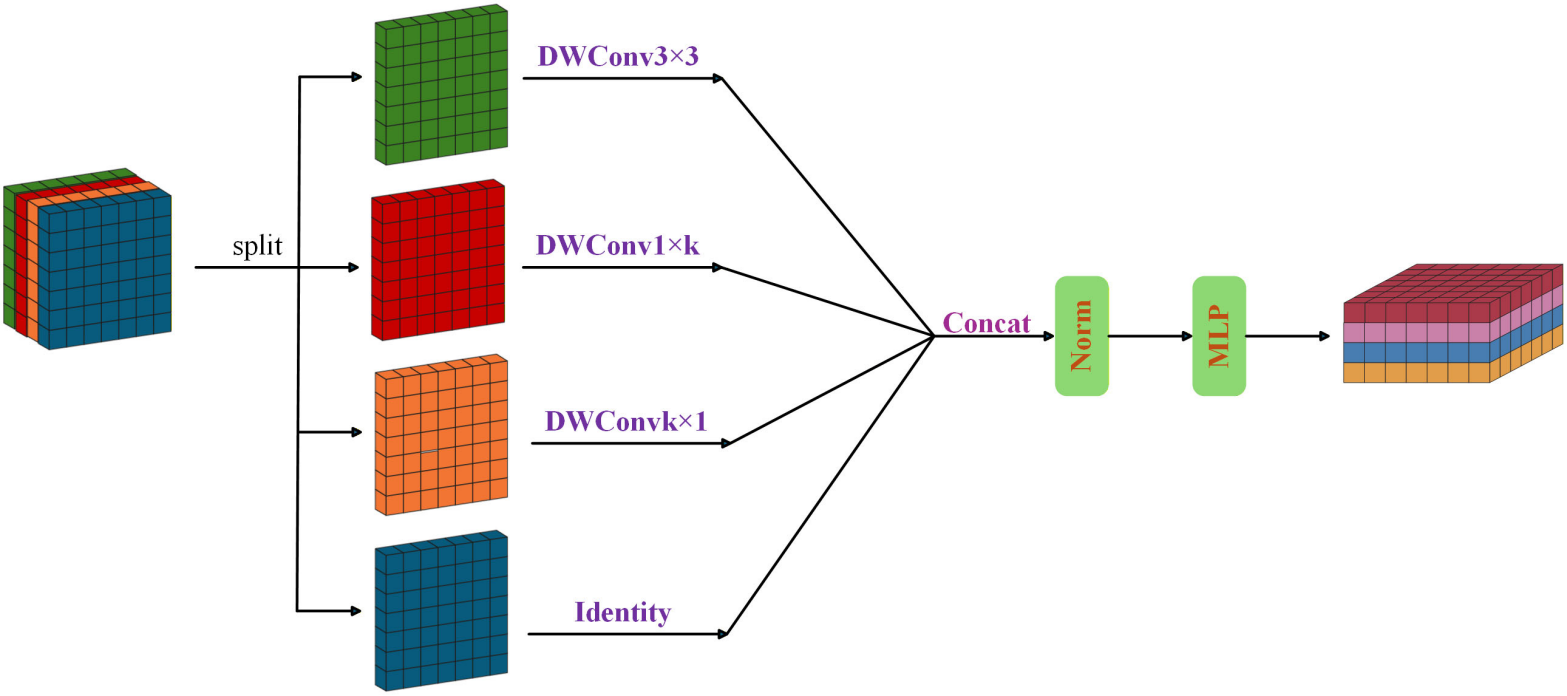
DWConv architecture diagram.

To significantly reduce memory access costs and computational complexity, thereby improving training and inference throughput, we replace the depthwise separable convolutions and pointwise convolutions in the feature focusing module with InceptionDWBlock. This module decomposes large-kernel depthwise convolutions along the channel dimension into four parallel branches to reduce computational complexity and memory access costs (Yu et al., 2024). **Figure 9** shows the architecture of InceptionDWBlock. As shown in the figure, this module comprises four branches: the small square kernel branch, which processes a portion of the channels using 3×3 kernels for depthwise convolution; the horizontal strip kernel branch, which processes a portion of the channels using 1×k kernels (where k=11 by default); the vertical strip kernel branch, which processes a portion of the channels using k×1 kernels (where k=11 by default); and the identity mapping branch, which applies no convolution transformation to the remaining channels, instead passing them through via an identity mapping. The input feature map is split into four groups along the channel dimension and forwarded into the corresponding branches for independent processing; finally, the outputs from each branch are concatenated along the channel dimension.

To validate the effectiveness of each component in FDPN, this paper conducts a systematic ablation study. By progressively introducing each core component, we quantitatively evaluate the independent contribution of each module to the model’s performance. The baseline uses the original Feature Pyramid Network. Building upon this, we sequentially introduce the Feature Focusing module (FocusFeature), replace the standard depthwise separable convolutions with InceptionDWConv, and implement a two-stage feature diffusion mechanism, ultimately forming the complete FDPN. The experimental configurations and results are shown in **Tables 2**, **3**.

**Table 2:** Self-comparison of FDPN parameters, GFLOPS, and MACs.

Model	Focus feature	InceptionDWConv	Two-stage diffusion	Parameter	GFLOPS	MACs
Baseline				4.05	8.7212	3.836
Exp-1	✓			7.38	17.514	8.492
Exp-2	✓	✓		9.97	24.836	12.071
Exp-3			✓	8.39	20.437	10.014
Full FDPN	✓	✓	✓	15.89	41.2668	20.303

**Table 3:** Self-comparison of precision, recall, F1 score, overall accuracy, and inference time for FDPN.

	Focus feature	InceptionDWConv	Two-stage diffusion	P(%)	R(%)	F1(%)	mAP@50(%)	Inference time(ms)
Baseline				92.60	89.70	91.13	93.15	39.18
Exp-1	✓			94.13	92.38	93.24	94.27	40.53
Exp-2	✓	✓		95.83	95.21	95.52	95.86	41.24
Exp-3			✓	94.87	93.54	94.20	94.93	40.89
Full FDPN	✓	✓	✓	97.49	97.63	97.51	97.15	43.50

## 4 Manuscript formatting experimental setup and analysis of results

### 4.1 Experimental evaluation criteria

To comprehensively quantify the segmentation performance of different networks, this study adopted a set of evaluation metrics widely used in the field of image segmentation, including Precision, Recall, F1 Score, mean Average Precision (mAP), and inference time. The definitions of these metrics are as follows: TP (True Positive) refers to the number of predicted instances correctly identified as positive (i.e., with IoU exceeding the predefined threshold); FP (False Positive) refers to the number of predicted instances incorrectly classified as positive; FN (False Negative) refers to the number of ground-truth instances that fail to be detected by the model; TN (True Negative) refers to the number of background regions correctly identified as negative. Precision reflects the proportion of samples correctly classified as positive out of all samples predicted as positive by the model. A high Precision value indicates that the model generates few false positive predictions, meaning most of the positively predicted instances are indeed true positives. However, a high Precision is often accompanied by an increase in the false negative rate. Therefore, relying solely on Precision is insufficient to comprehensively evaluate model performance; it must be considered in conjunction with Recall to achieve an appropriate balance between false positive suppression and true positive coverage. The principle is as shown in Equation (8).

(8) $$\mathrm{Pr}\text{ecision=}\frac{TP}{TP+FP}$$

Recall is another important metric for evaluating the performance of classification models, and it is particularly critical in scenarios where minimizing false negatives is of primary concern, such as instance segmentation tasks. Recall measures the proportion of ground-truth positive instances that are correctly identified by the model. The principle is as shown in Equation (9).

(9) Recall=TPTP+FN

Recall quantifies the extent to which a model captures positive instances, reflecting how many ground-truth positive instances the model successfully identifies. A higher Recall indicates that the model successfully identifies a greater proportion of true positive instances; however, this may come at the cost of an increased false positive rate, as the decision boundary becomes more inclusive. In applications such as potato yield estimation, where missing a true positive instance directly affects counting accuracy, Recall becomes critically important, as failing to detect a true positive instance may lead to significant errors in downstream tasks. Furthermore, there exists an inherent tension between Recall and Precision. Improving a model’s Recall typically reduces Precision, as capturing more positive instances may result in some negative instances being incorrectly predicted as positives. Therefore, in practical applications, one should consider both Recall and Precision based on the task’s characteristics, and select an appropriate decision threshold or optimization strategy to strike a balance between these two metrics.

The F1 Score represents the harmonic mean of Precision and Recall. The principle is as shown in Equation (10).

(10) $$F_{1\text{score}}=\frac{2\times \mathrm{Pr}\text{ecision}\times \text{Recall}}{\mathrm{Pr}\text{ecision}+\text{Recall}}$$

Mean Average Precision (mAP) is used as a metric to evaluate the detection and segmentation accuracy across the entire dataset. mAP is computed as the mean of the Average Precision (AP) values across all object categories, and a higher mAP value indicates superior overall detection and segmentation performance. P represents Precision, and AP_i_ denotes the Average Precision for the i-th category; T denotes the total number of object categories in the dataset. The principle is as shown in Equations (11, 12).

(11) $$AP_{\text{i}}=\frac{P}{N}$$

(12) $$\text{mAP=}\frac{\sum_{\text{i}=1}^{T}AP_{I}}{T}\times 100\%$$

Inference time refers to the time required for a model to complete inference on a single input image. This time depends on both the model’s computational complexity and the hardware platform employed. Consequently, inference time serves as a practical indicator of the model’s parameter count, architectural depth, and operational complexity. In the context of this study, inference time is particularly relevant as it directly determines whether the model meets the real-time processing requirements of the potato harvester conveyor belt system.

**Figure 10:**
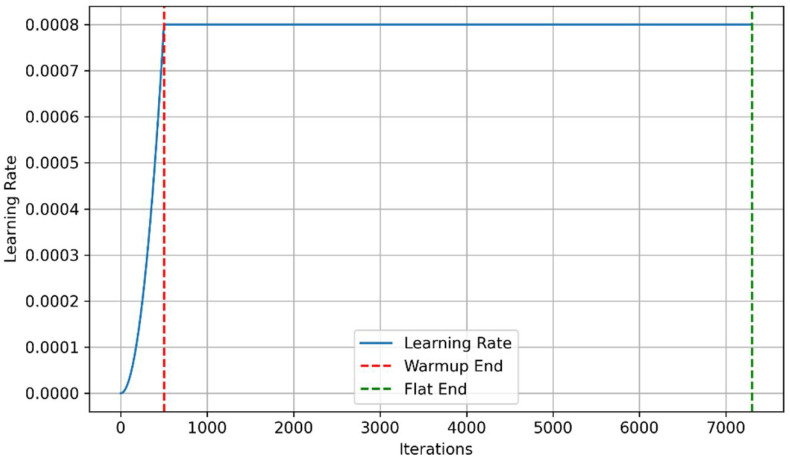
Flat cosine learning rate schedule adopted during training.

The model was optimized using a Flat Cosine Learning Rate Schedule (**Figure 10**). The schedule begins with a linear warmup over 500 iterations, ramping the learning rate from 0 to a peak of 8×10^−4^, followed by a flat plateau sustained until iteration 7,200. This configuration ensures training stability in the early stage while maintaining a consistently high learning rate throughout the main training phase.

**Figure 11:**
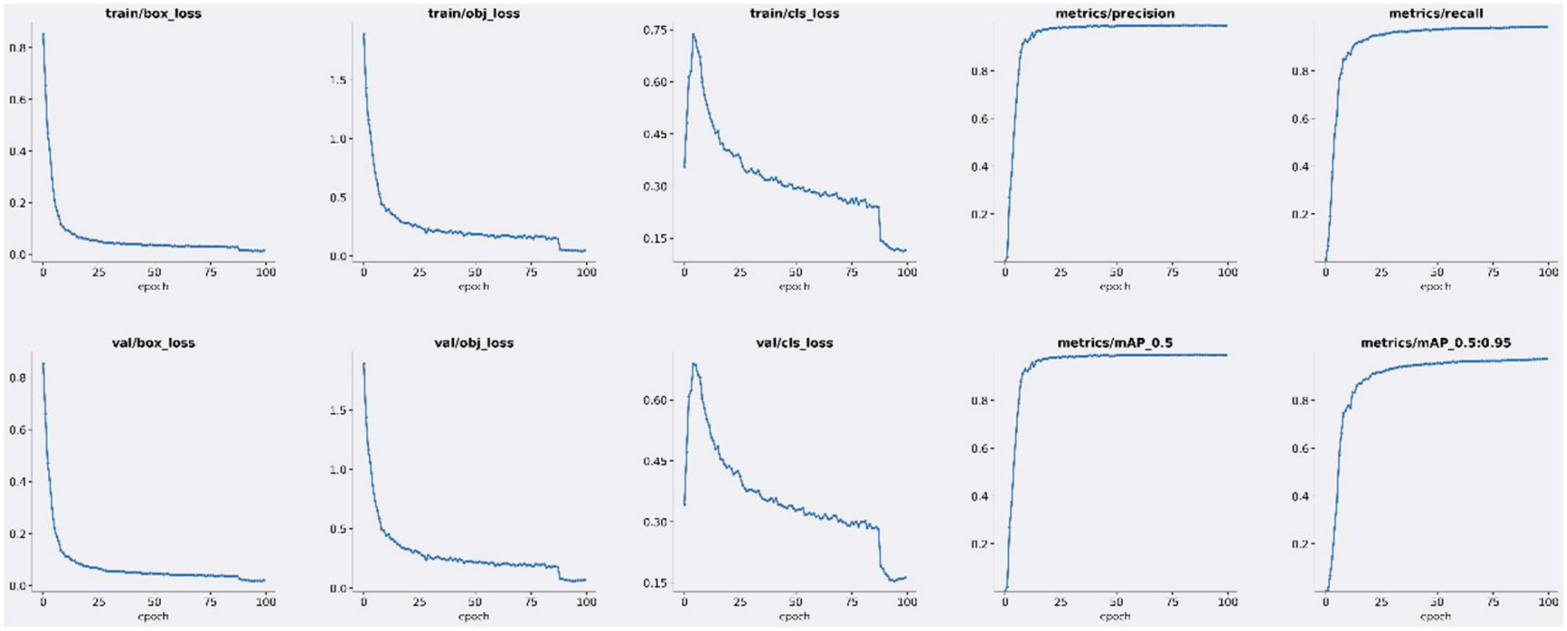
Training loss and evaluation metrics curves over epochs.

**Figure 11** illustrates the variation of training and validation metrics over the course of model training. As shown in the loss curves, both the training and validation losses, including box_loss, obj_loss, and cls_loss, exhibit a rapid decline in the early stage (epochs 0–30), followed by gradual convergence and stabilization. This indicates that the model effectively captures the key features of the target objects during the initial training phase. The close alignment between training and validation loss curves throughout the entire training process suggests that the model generalizes well to unseen data without overfitting.

**Figure 12:**
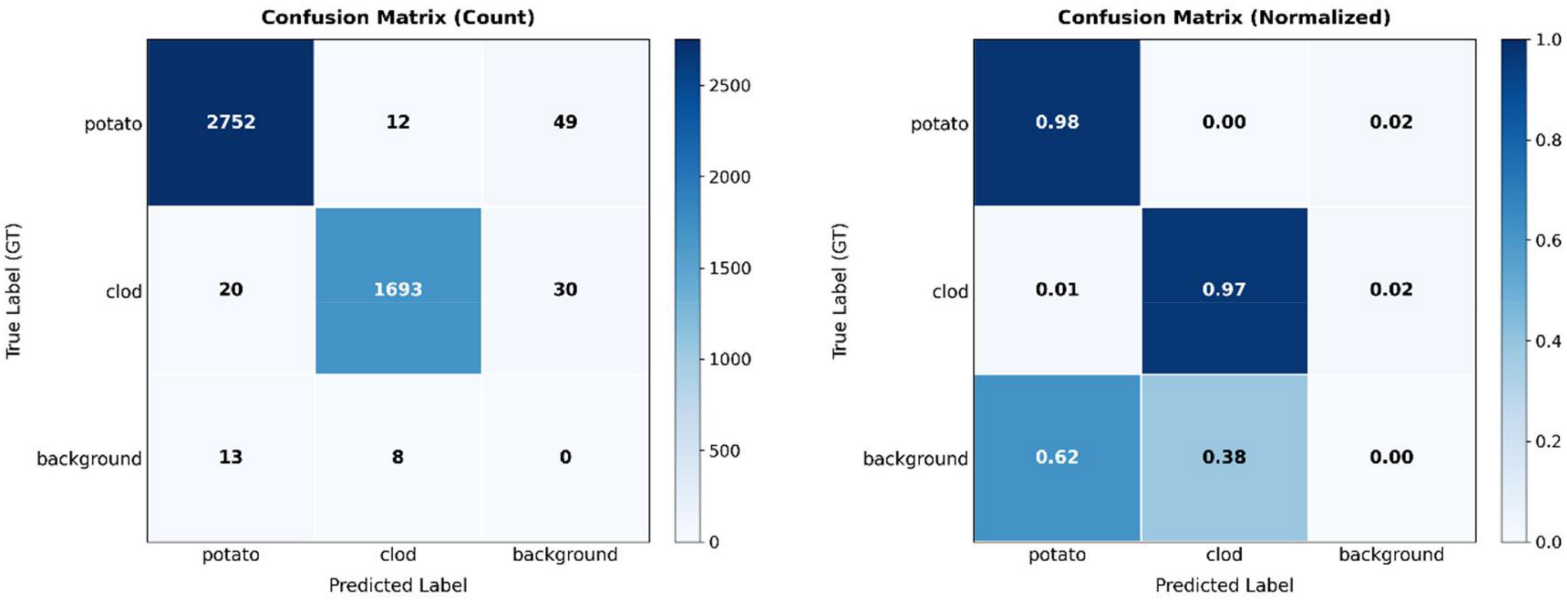
Confusion matrix of detection results (potato & clod).

To further evaluate the classification performance of the model on potato and clod categories, the confusion matrix of Mask R-CNN on the validation set is presented in **Figure 12**. As shown, the model correctly detected 2,752 potato instances with only 49 missed detections and 12 misclassified as clod. For the clod category, 1,693 instances were correctly detected, with 30 missed detections and 20 misclassified as potato. In the normalized confusion matrix, the correct recognition rates for both potato and clod reach 0.98, with negligible inter-class confusion. These results demonstrate that the proposed model can effectively distinguish between morphologically similar potato and clod objects, achieving high classification accuracy and strong robustness, which validates the effectiveness of the proposed detection approach.

### 4.2 Self-comparison of DFINE-PFM model experiments

To evaluate each module’s contribution, ablation studies were conducted using DFINE as the baseline with modules introduced incrementally.

Introducing FDPN improved Precision from 92.60% to 95.39%, Recall from 89.70% to 95.30%, with F1 and mAP gains of 4.69 and 2.70 percentage points respectively. The three-scale parallel input structure and multi-branch receptive field design enable simultaneous capture of high-resolution local details and global semantic information, mitigating cross-scale feature degradation inherent in traditional FPNs. Parameter count increased from 4.05×10^6^ to 4.73×10^6^ with GFLOPs rising from 8.72 to 11.57, remaining within acceptable computational bounds.

Further incorporating IDWB achieved Precision of 97.49%, Recall of 97.63%, and mAP of 97.15%, validating its advantage in collaborative multi-scale feature extraction through four parallel depthwise convolution branches. However, parameters increased substantially to 15.89×10^6^ with GFLOPs reaching 41.27, introducing significant computational redundancy.

Replacing RepNCSPELAN4 with MSBlock_Plus resolved this bottleneck by reducing parameters from 15.89×10^6^ to 6.46×10^6^ (−59.3%) and GFLOPs from 41.27 to 21.35 (−48.3%), while simultaneously improving Precision, Recall, F1, and mAP to 98.60%, 98.30%, 98.40%, and 97.45%, achieving dual optimization of accuracy and efficiency.

Finally, introducing PSConv to replace standard 1×1 convolutions in backbone lower layers added only 0.31×10^6^ parameters while achieving the best overall performance: Precision 98.80%, Recall 98.70%, F1 98.75%, and mAP 98.03% at 43.41 ms inference time, representing gains of 6.20 and 5.60 percentage points in Precision and mAP over the baseline.

The four modules exhibit clear complementarity: FDPN establishes multi-scale fusion foundations, IDWB enhances fine-grained boundary detection, MSBlock_Plus reduces model complexity while improving accuracy, and PSConv refines small object localization. Their progressive integration delivers consistent performance gains while maintaining real-time capability, demonstrating strong potential for practical deployment.

The self-comparisons of the models are shown in **Tables 4**, **5**.

**Table 4:** Self-comparison of parameters, GFLOPS, and MACs for the model’s backbone network.

Model	Parameter	GFLOPS	MACs
DFINE	4.05	8.7212	3.836
DFINE+FDPN	4.73	11.5653	5.241
DFINE+FDPN+IDWB	15.89	41.2668	20.303
DFINE+FDPN+IDWB+MSBlock_Plus	6.46	21.3515	8.998
DFINE+FDPN+IDWB+MSBlock_Plus+PSConv	6.77	24.4813	10.331

**Table 5:** Self-comparison of precision, recall, F1 score, overall accuracy, and inference time for the model’s backbone network.

Configuration	P (%)	R (%)	F1 (%)	mAP@50 (%)	Inference time(ms)
DFINE	92.60	89.70	91.13	93.15	39.18
DFINE+FDPN	95.39	94.30	95.82	95.85	42.45
DFINE+FDPN+IDWB	97.49	97.63	97.51	97.15	43.50
DFINE+FDPN+IDWB+ MSBlock_Plus	98.60	98.30	98.40	97.45	43.29
DFINE+FDPN+IDWB+ MSBlock_Plus+PSConv	98.80	98.70	98.75	98.03	43.41

**Figure 13:**
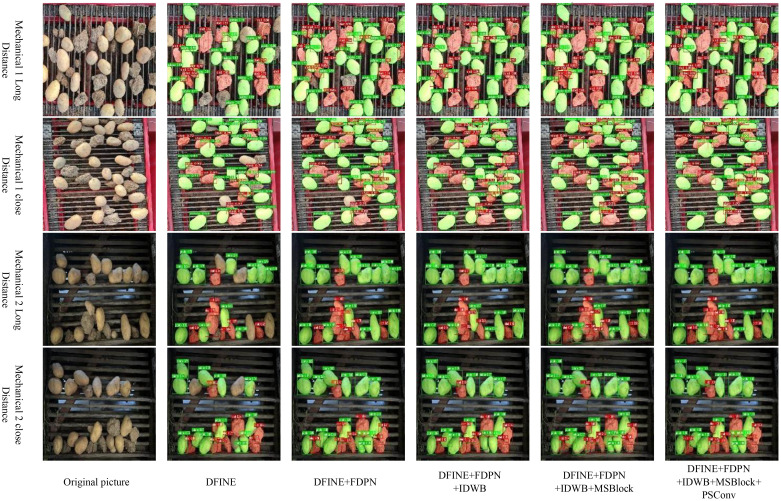
Segmentation results for the five models (potatoes and soil clumps are shown in green and red, respectively).

**Figure 14:**
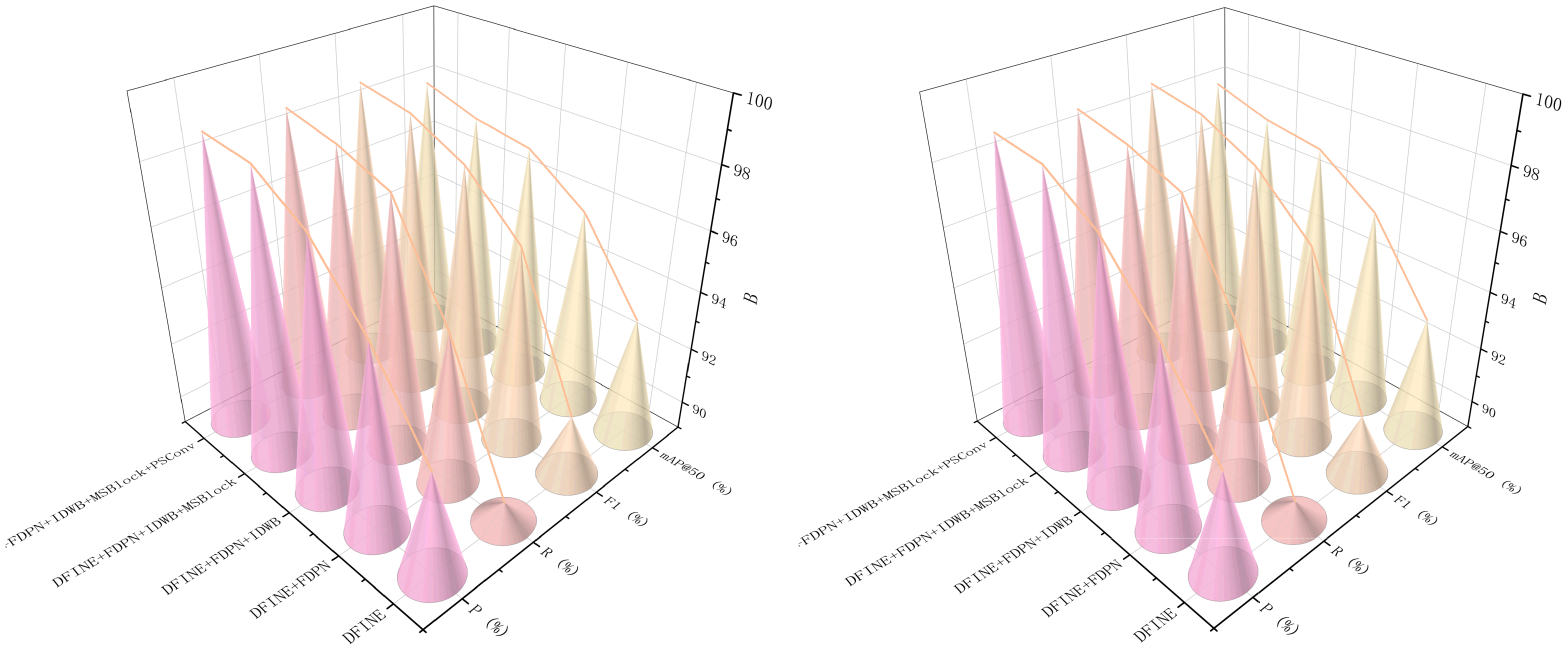
Visualization of model performance (ablation studies).

To provide a more visual and intuitive illustration of how the progressive introduction of each proposed module affects segmentation performance, the final segmentation results for each model and their comparisons are shown in **Figures 13**, **14**.

### 4.3 Comparison of DFINE-PFM model experiments with those of other models

To comprehensively evaluate the performance of the DFINE-PFM segmentation model, we compared its segmentation results with those of five state-of-the-art segmentation models: Mask R-CNN (He et al., 2017), Mask RT-DETR (Fang et al., 2021), Mask DINO (Li et al., 2023), QueryInst (Zhao et al., 2024), and YOLOv11-seg (Khanam and Hussain, 2024). All six segmentation models utilized the same dataset and loss function as detailed in the preceding sections, and the evaluation metrics introduced in Section 4.1 were applied to evaluate the segmentation models. To ensure a fair and controlled experimental comparison, the YOLO series models were configured with their official default hyperparameters provided by Ultralytics, while the RT-DETR series used the same hyperparameters as the proposed method in this study; neither utilized pre-trained weights. For full transparency and to ensure that performance differences reflect architecture rather than training budget, all models were trained and evaluated under a unified protocol: the identical train/validation/test split, the same input resolution, the same data-augmentation pipeline, the same number of training iterations, and the same optimizer settings, with no pre-trained weights for any model, on the same NVIDIA RTX 4090 platform. The complete per-model configuration—input size, augmentation, epochs, optimizer, learning rate, backbone, use of pre-trained weights, and hardware summarized in the training-settings table (**Table 6**) to allow direct reproducibility. The experimental results are shown in **Table 3**. In terms of the primary segmentation performance metrics F1 Score and mAP@50, DFINE-PFM outperformed other detectors by 1.21–5.36% and 0.59–8.30%, respectively, achieving maximum values of 98.75% and 98.03%. This indicates that DFINE-PFM can detect objects more accurately, effectively reduce false negatives in small instance detection scenarios, and achieve the highest overall segmentation performance among all compared models in terms of both stability and accuracy. In terms of computational efficiency, DFINE-PFM achieves the second lowest computational complexity among all compared models, surpassed only by YOLOv11-seg, while maintaining competitive inference speed.

**Table 6:** Training and evaluation configurations of all compared models.

	DFINE-PFM	Yolov11-seg	QueryInst	Mask DINO	Mask RT-DETR	Mask R-CNN
Backbone	HGNetV2	YOLOv11(CSP)	ResNet-50-FPN	ResNet-50	HGNetV2-M	ResNet-50-FPN
Pre-trained weights	None	None	None	None	None	None
Training input size	640×640	640×640	1333×800	1333×800	640×640	1333×800
Train batch size	16	16	16	16	16	16
Training epochs	160	160	160	160	160	160
Optimizer	AdamW	SGD	AdamW	AdamW	AdamW	SGD
Data augmentation	Mosaic + MixUp + Directional denoise	Mosaic + MixUp + Directional denoise	Mosaic + MixUp + Directional denoise	Mosaic + MixUp + directional denoise	Mosaic + MixUp + Directional denoise	Mosaic + MixUp + Directional denoise

The comparison results of model parameters are shown in **Table 7**.

**Table 7:** Comparison of model performance.

Model	P(%)	R(%)	F1(%)	mAP@50(%)	Inference time(ms)	Parameter	GFLOPS	MACs
Yolov11-seg	96.79	97.68	97.21	96.01	41.38	2.84	4.86	2.43
QueryInst	96.61	95.90	96.25	95.40	109.94	172.45	50.59	25.30
Mask DINO	94.03	92.80	93.39	89.73	111.51	43.78	98.95	49.48
Mask RT-DETR	96.61	94.43	95.51	94.24	77.46	25.06	68.00	34.00
Mask R-CNN	97.81	97.86	97.54	97.44	97.99	43.98	50.29	25.30
DFINE-PFM	98.80	98.70	98.75	98.03	43.41	6.77	24.48	10.33

**Figure 15:**
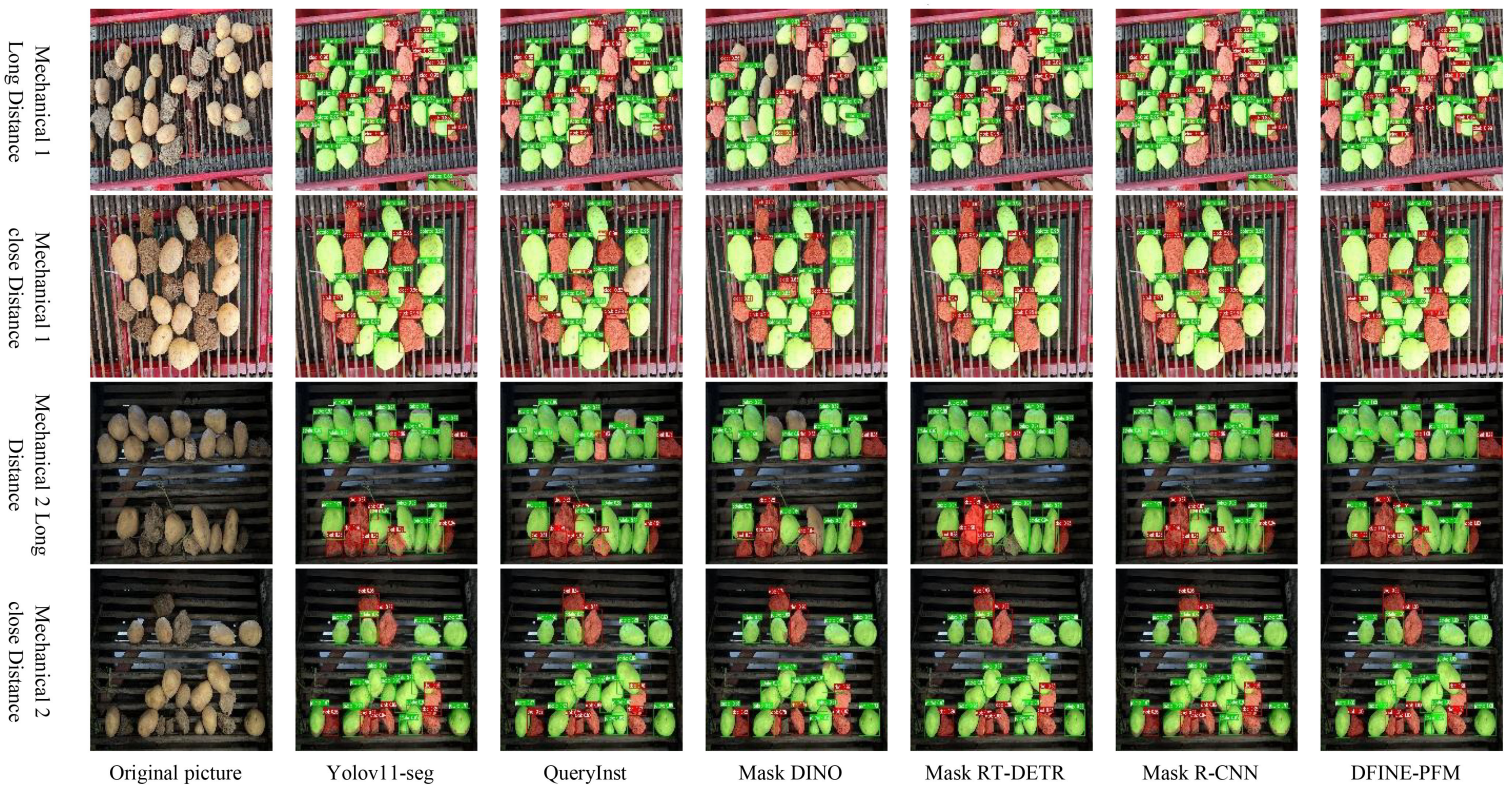
Detection in different scenarios.

To verify the actual performance of DFINE-PFM in various scenarios, qualitative evaluation experiments were conducted on representative test samples, as shown in **Figure 15**. The results indicate that, compared to other models, the proposed model demonstrates greater sensitivity to target regions and stronger robustness against background clutter, inter-instance occlusion, and soil adhesion in real-world conveyor belt scenarios, thereby confirming its practical applicability in complex agricultural environments.

**Figure 16:**
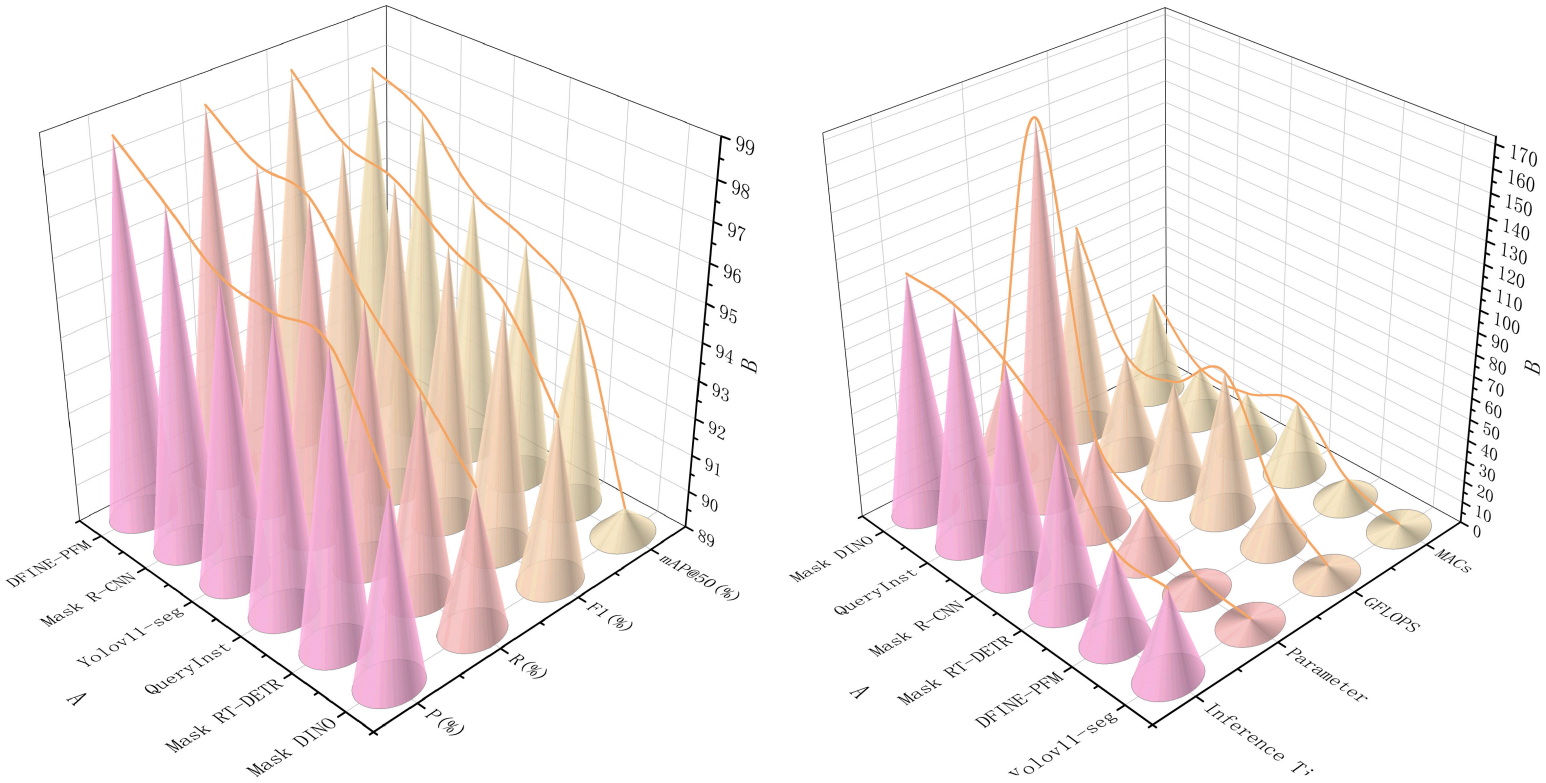
Visualization of model performance (comparative experiment).

To further facilitate a holistic and multi-dimensional comparison of each model’s segmentation performance across all evaluation metrics, the experimental results were visualized as radar charts to intuitively compare the models’ performance, as shown in **Figure 16**.

## 5 Bench-top experiment setup and results

### 5.1 DFINE-PFM deployment on Jetson Orin Nano for potato segmentation and tracking

To simulate the deployment performance of the DFINE-PFM instance segmentation algorithm on edge devices and systematically evaluate its practical value in real-world operational scenarios, this paper establishes an integrated segmentation-tracking experimental platform for potatoes and soil clods. The perception module of the system consists of an Intel RealSense D456 depth camera mounted above the platform, which continuously captures RGB images of the conveyor belt’s working area and transmits them to the segmentation and tracking modules for subsequent processing.

In actual production environments, due to the continuous motion of the conveyor belt, the detection confidence of target instances frequently degrades transiently owing to mutual occlusion, motion blur, and boundary truncation at the edges of the field of view. Traditional multi-object tracking algorithms (such as SORT and DeepSORT) typically discard low-confidence detections entirely, leading to trajectory fragmentation and frequent identity switches (ID switches), making it difficult to meet the requirements of industrial scenarios for tracking continuity and counting accuracy. To address this, this paper introduces the ByteTrack tracking algorithm. Based on the core assumption that “low-confidence detections may still contain real targets,” this algorithm classifies detection results into two groups—high-confidence and low-confidence—based on confidence scores: first, it performs primary association using high-confidence detections and existing trajectories; then, it performs secondary association between trajectories not matched in the primary association and low-confidence detections, thereby effectively recovering real targets whose detection confidence was suppressed due to transient occlusion or shape deformation (Zhang et al., 2022). Furthermore, ByteTrack relies solely on Kalman filter-based motion information for data association, eliminating the need for additional appearance feature extraction networks. It offers excellent real-time performance and lightweight characteristics, making it well-suited for the computational constraints of industrial deployments. The mathematical formula underlying the ByteTrack tracking algorithm is as follows:

Let the set of detection results output by the t-th frame detector be denoted by, where bi,si and ci represent the bounding box, confidence score, and class, respectively. The core idea of ByteTrack is to divide the detection results into two groups based on high and low confidence thresholds $\;\tau_{h}$and $\;\tau_{l}(\tau_{l}<\tau_{h})$. The principle is as shown in Equation (13).

(13) $$D_{t}^{high}\;=\{d_{i}|{\text{s}}_{i}\geq \tau_{\text{h}}\;\},D_{\text{t}}^{\text{low}}\;=\{d_{i}|\tau_{l}\leq {\text{s}}_{i}\leq \tau_{\text{h}}\}$$

The predicted bounding box for the current frame is obtained via Kalman filtering, and the association cost between the trajectory and the detection is calculated based on the IoU distance: The principle is as shown in Equation (14).

(14) $$C_{ij}=1-IoU({\hat{b}}_{j},b_{i})$$

Data linking was then performed using a two-stage Hungarian matching algorithm: The principle is as shown in Equations (15, 16).

(15) $$M^{\left(1\right)}=H(T_{t-1},D_{t}^{high};\epsilon_{1})$$

(16) $$M^{\left(2\right)}=H(T_{remain},D_{t}^{low};\epsilon_{2})$$

Building upon the tracking module, this paper further introduces a virtual counting line mechanism to enable target counting. When a target’s center of mass first crosses the predefined counting line, the system records its category and triggers a counting event; thereafter, the target’s trajectory is no longer included in subsequent recognition, segmentation, and counting operations, thereby preventing double counting. Combined with ByteTrack’s stable trajectory identity, this mechanism enables stable and accurate real-time counting during the continuous movement of potatoes.

Here, H(•)denotes the Hungarian matching operator, ϵ1, ϵ2represents the IoU association threshold for the corresponding stage, and denotes the set of trajectories that failed to establish a stable association in the first stage. The first stage utilizes high-confidence detection to establish stable associations, while the second stage employs low-confidence detection to recover true targets that were underestimated due to occlusion, motion blur, or partial loss of view. This significantly reduces the identity switching rate and improves tracking continuity.

### 5.2 Evaluation of potato segmentation and tracking accuracy

**Figure 17:**
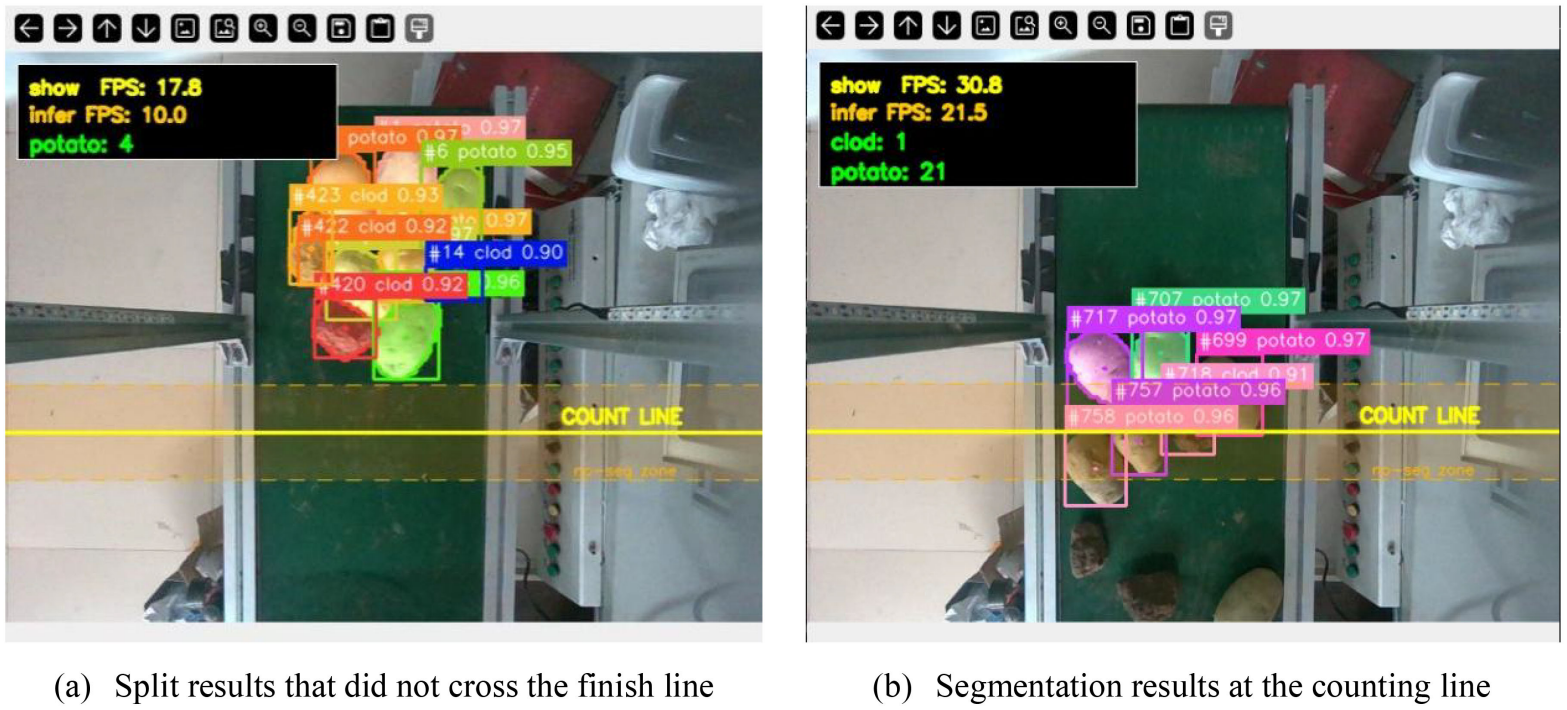
Actual segmentation results from the experimental platform. **(a)** Split results that did not cross the finish line. **(b)** Segmentation results at the counting line.

In this method, a depth camera captures real-time images of potatoes and clumps of soil on the conveyor belt, and an improved DFINE algorithm is used to segment the captured images; this segmentation helps identify target objects within the target area. **Figure 17** shows the results of the actual segmentation on the experimental platform.

As shown in the figure, the FPS remains stable at around 20 after deploying the DFINE-PFM algorithm, while the operating speed range of the conveyor belt on a conventional potato harvester is 0.1–0.6 m/s, with an optimal operating speed of 0.4 m/s. The experimental results from the test platform indicate that the algorithm can quickly and accurately segment potatoes from clods of soil. Therefore, the deployment of the DFINE-PFM algorithm is fully suitable for traditional potato harvesters.

To further validate the performance of DFINE-PFM under actual operating conditions, we conducted quantitative benchtop experiments on the Jetson Orin Nano platform using the conveyor belt and RealSense D456 camera described in Section 5.1. In each trial, a set number of objects—six potatoes and three soil clods, varying in size and soil coverage—were placed on the conveyor belt and passed through the detection zone; each condition was repeated five times, with samples rearranged between trials, and all metrics were averaged.

Under normal operating conditions with a conveyor speed of 0.4 m/s, the system achieved a counting accuracy of 92% for potatoes and 94% for soil clods (93% overall), with a missed detection rate of 12% and a false detection rate of 4%. These results demonstrate the system’s ability to reliably identify and count potatoes and soil clods on the conveyor belt, a capability crucial for accurate yield estimation and automated clod removal.

To evaluate sustained real-time performance, the system was operated continuously for 10 minutes while instantaneous frame rates were recorded. The frame rate remained stable between 15 and 31 FPS (ranging from a minimum of 15.4 FPS to a maximum of 30.8 FPS). This confirms that the reported throughput reflects a stable, long-term operating state rather than transient peaks, and demonstrates the system’s ability to consistently meet detection requirements for conveyor speeds ranging from 0.1 to 0.6 m/s.

## 6 Discussion and conclusions

### 6.1 Discussion

The DFINE-PFM proposed in this paper is an end-to-end, single-stage network that simultaneously detects and segments potato and soil clod regions. Compared to traditional multi-stage detection and segmentation methods, DFINE-PFM unifies detection and segmentation within a single framework. Its training process eliminates the need for multi-step cascaded training, resulting in a more streamlined optimization pipeline, and it demonstrates significant advantages in both segmentation accuracy and inference speed (see **Table 4**).

The core architecture of DFINE-PFM consists of four components. The first is the Feature Focused Diffusion Pyramid Network (FDPN). Based on a three-scale parallel feature input structure and a multi-branch receptive field design in the feature focusing module, the FDPN enables the simultaneous extraction of target features at different scales, enhancing the network’s ability to handle complex scenarios such as scale differences, occlusion, and stacking between potatoes and soil clods. Furthermore, the FDPN effectively balances high-resolution local spatial details with low-resolution global semantic information, effectively mitigating the information loss typically encountered during cross-scale propagation in traditional FPNs. The second is the InceptionDWBlock (IDWB). By decomposing large-kernel depthwise convolutions along the channel dimension into four parallel branches—small square kernels, horizontal strip kernels, vertical strip kernels, and identity mappings—IDWB simultaneously captures both the irregular contours and surface texture information of potato tubers within a single module. Compared to traditional combinations of independent depthwise convolutions, it offers a distinct advantage in the collaborative extraction of multi-scale features, significantly enhancing the network’s ability to perceive challenging samples such as the boundaries of buried potato tubers. The third is the MSBlock_Plus feature enhancement module. Building upon the synergistic design of an inverted bottleneck structure and a grouped accumulation mechanism inherited from the original MSBlock, MSBlock_Plus introduces four targeted improvements: a heterogeneous multi-scale kernel configuration [1, 3, 5], a bidirectional progressive feature aggregation mechanism, an adaptive branch importance gating strategy, and a module-level residual connection. Through these enhancements, MSBlock_Plus achieves comprehensive multi-granularity feature capture—ranging from fine-grained epidermal surface textures to coarse-grained morphological contours—with limited computational overhead. The heterogeneous kernel configuration eliminates inter-branch feature redundancy while enabling complementary perception of potato surface details (3×3) and tuber contour curvature (5×5). The bidirectional aggregation mechanism transforms the asymmetric unidirectional information flow of the original MSBlock into a fully connected inter-branch information sharing paradigm, ensuring that both shallow fine-grained and deep semantic representations are accessible to all branches. The adaptive gating strategy dynamically reweights branch contributions based on input content, replacing the fixed equal-weight aggregation of the original design. The module-level residual connection safeguards non-directional semantic information—such as color statistics and global context—against loss during the 2:1 channel compression at encoder fusion nodes. Collectively, these improvements effectively suppress the “contaminated spatial information” issue commonly found in Res2Net-like architectures while further enhancing the ability to distinguish multi-scale potato targets, achieving dual optimization of segmentation accuracy and computational efficiency. The fourth is the Windmill-shaped Convolution (PSConv). PSConv replaces the standard 1×1 convolutions in the lower layers of the backbone network. By generating a windmill-shaped receptive field through four-directional asymmetric padding, it better matches the Gaussian spatial distribution of pixels in small and low-contrast targets. With only a marginal increase in parameters, it effectively suppresses interference from background noise and irrelevant semantic information.

**Figure 18:**
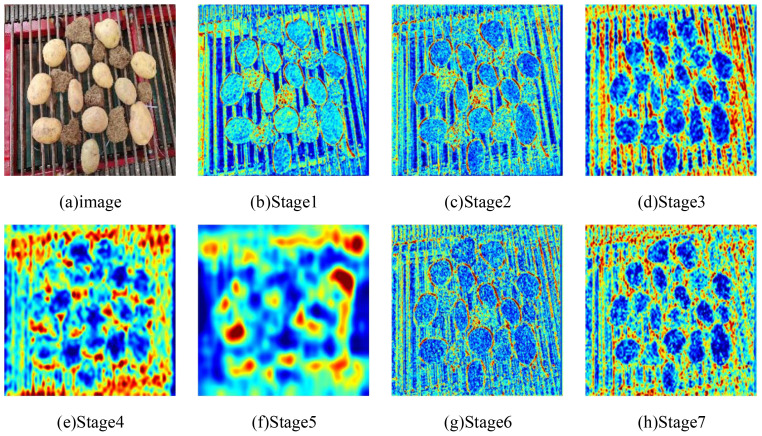
Visualization of feature maps extracted at each stage of DEIM-PFM. **(a)** Image. **(b)** Stage1. **(c)** Stage2. **(d)** stage 3. **(e)** Stage4. **(f)** Stage5. **(g)** Stage6. **(h)** Stage7.

Therefore, as shown in Section 4.3, the DFINE-PFM proposed in this paper demonstrates higher segmentation accuracy than other comparison methods in the complex operational scenarios of a potato harvester conveyor belt (see **Table 4**). Additionally, **Figure 18** presents visualizations of the feature maps output at seven stages in DFINE-PFM. **Figures 18B, C** correspond to the feature maps output by the shallow layers of the backbone network, Stages 1 and 2. It can be observed that at this stage, the network primarily responds to low-level texture and edge information in the image; the structural texture of the conveyor belt grid is significantly activated, and the boundary contours of potatoes and soil clods have begun to emerge, though the distinction between the target regions and the background is not yet clear. **Figures 18D, E** correspond to the feature maps output by the intermediate stages (Stage 3 and Stage 4). As the network depth increases, feature representations gradually transition from low-level textures to high-level semantics. The network begins to focus its attention on the target regions of the potatoes and soil clods, while the interference response from the background grid weakens. The instance-level contours of each target become increasingly distinct, indicating that the network has developed a certain ability to distinguish targets. **Figure 18F** corresponds to the output feature map of the deep stage (Stage 5), i.e., the output of the TransformerEncoderBlock. At this stage, the global self-attention mechanism establishes long-range contextual relationships between potato instances in the image. Feature activation is highly concentrated on the target as a whole, while background responses are significantly suppressed, demonstrating pronounced high-level semantic concentration. **Figures 18G, H** correspond to the output feature maps of the encoder fusion stages (Stages 6 and 7), representing the first multi-scale fusion output of the FocusFeature_IDWB module and the feature enhancement output of MSBlock_Plus, respectively. It can be observed that at this stage, through the cross-scale collaborative extraction achieved by IDWB’s multi-branch parallel depthwise convolutions, as well as the comprehensive multi-granularity feature enhancement provided by MSBlock_Plus—enabled by its heterogeneous kernel configuration [1, 3, 5], bidirectional progressive aggregation, adaptive branch gating, and module-level residual connection—the network simultaneously captures both high-resolution local boundary details (potato epidermal textures and micro-crack edges of soil clods) and low-resolution global semantic information (overall morphological contours of potato tubers). Compared to the original MSBlock, the bidirectional aggregation mechanism enables shallow branches to benefit from the semantic representations of deep branches, resulting in more discriminative boundary activations, while the adaptive gating dynamically amplifies the most informative scale features for each spatial region. The instance boundary responses for potatoes and soil clods are particularly distinct, providing highly discriminative multi-scale feature representations that enable the subsequent DFINESegTransformer decoder to achieve precise instance-wise contour segmentation.

### 6.2 Conclusion

This paper proposes an improved DFINE algorithm for the recognition and segmentation of potatoes and soil clods on the conveyor belt of a potato harvester, which can be applied to potato yield estimation and sorting for impurity removal. By integrating the Feature Focused Diffusion Pyramid Network (FDPN), InceptionDWBlock, PSConv, and MSBlock_Plus to optimize the model, segmentation performance has been significantly enhanced. The improved algorithm increased the mean average precision (mAP) for potato and soil clod segmentation by 5.60 percentage points, achieving a notable peak during the validation phase of the ablation study. Compared to the original DFINE network, the improved model demonstrates significant enhancements across key metrics. Through a detailed comparison with state-of-the-art instance segmentation algorithms such as YOLOv11-seg, QueryInst, Mask DINO, Mask RT-DETR, and Mask R-CNN, experimental results indicate that the improved DFINE model demonstrates superior overall segmentation performance, achieving a Precision (P) of 98.8%, representing a 6.2 percentage point improvement. Furthermore, the model achieves an inference time of 43.41 ms, effectively meeting real-time operational requirements.

Although the algorithm demonstrates superior performance, it is constrained by the limited diversity and potential distribution bias of the training dataset. Scenarios and environments insufficiently covered by the training data may negatively affect the algorithm’s generalization ability. Experiments conducted on the experimental platform confirm the algorithm’s applicability to practical deployment scenarios. Real-time segmentation of potatoes and soil clods using a depth camera enables the extraction of camera-to-object distance information, offering substantial potential for real-time yield estimation and automated soil removal applications. The introduction of the tracking algorithm and counting lines significantly reduces false identifications of potatoes and soil clods, which has a considerable impact on counting accuracy and real-time yield estimation. The evaluation results from the experimental platform highlight the model’s substantial potential for segmenting potatoes and soil clods on conveyor belts.

In future work, the further integration of the improved DFINE algorithm with feature-focused diffusion networks presents promising research directions. This combination can be leveraged to enhance target detail representation, minimize the loss of essential features, and reduce the number of parameters and computational costs. Optimizing the algorithm to reduce computational demands without compromising segmentation performance remains a key future direction, which could make DFINE-PFM more suitable for deployment in resource-constrained real-time applications.

## Data Availability

The raw data supporting the conclusions of this article will be made available by the authors, without undue reservation.
